# Discordance between cystatin C–based and creatinine-based estimated glomerular filtration rate and health outcomes in adults: a systematic review and meta-analysis

**DOI:** 10.1093/ckj/sfaf003

**Published:** 2025-01-08

**Authors:** Qiaoling Liu, Paul Welsh, Carlos Celis-Morales, Frederick K Ho, Jennifer S Lees, Patrick B Mark

**Affiliations:** School of Cardiovascular and Metabolic Health, University of Glasgow, Glasgow, UK; Department of Medicine Solna, Karolinska Institute, Stockholm, Sweden; School of Cardiovascular and Metabolic Health, University of Glasgow, Glasgow, UK; School of Cardiovascular and Metabolic Health, University of Glasgow, Glasgow, UK; Human Performance Lab, Education, Physical Activity and Health Research Unit, Universidad Católica del Maule, Maule, Chile; High-Altitude Medicine Research Centre (CEIMA), Universidad Arturo Prat, Iquique, Chile; School of Health and Wellbeing, University of Glasgow, Glasgow, UK; School of Cardiovascular and Metabolic Health, University of Glasgow, Glasgow, UK; School of Cardiovascular and Metabolic Health, University of Glasgow, Glasgow, UK

**Keywords:** adult, cystatin C, eGFR discordance, health outcome, serum creatinine

## Abstract

**Background:**

The intra-individual difference in cystatin C–based and creatinine-based estimated glomerular filtration rate (eGFRcys and eGFRcr, respectively), i.e. eGFR discordance, has recently been demonstrated to have prognostic implications. eGFR discordance was associated with mortality, cardiovascular and renal outcomes. We present a systematic review and meta-analysis to summarize the existing literature.

**Methods:**

We searched PubMed, Embase and MEDLINE up to 28 April 2024 for cohort and cross-sectional studies in English reporting the association of eGFR discordance with mortality, cardiovascular and renal outcomes. The quality of studies was evaluated by Risk Of Bias In Non-randomized Studies—of Exposure (ROBINS-E) form. Data from studies were extracted to a pre-defined table and pooled using a random-effects model. Stratified and sensitivity analyses were performed.

**Results:**

A total of 1489 studies were initially identified, of which 18 studies with longitudinal or cross-sectional designs were included, with a sample size between 373 and 363 494 people. In general, the risk of bias was graded as “low“ or “some concerns”. eGFR was mainly calculated using Chronic Kidney Disease Epidemiology Collaboration equations, while a few studies applied other equations. An eGFR discordance featuring lower eGFRcys, e.g. eGFRcys ≤60% of eGFRcr, or eGFRcys-eGFRcr ≤–15 mL/min/1.73 m^2^, was consistently associated with higher mortality and elevated risk of cardiovascular and renal outcomes. People with lower eGFRcys have a 58% greater risk of mortality [hazard ratio (HR) = 1.58, 95% confidence interval (CI) 1.42, 1.76] and 32% greater risk of cardiovascular events (HR = 1.32, 95% CI 1.25, 1.39). People with higher eGFRcys have a 39% lower risk of mortality (HR = 0.61, 95% CI 0.52, 0.70) and 29% lower risk of cardiovascular events (HR = 0.71, 95% CI 0.62, 0.81). No meta-analysis for renal outcomes was conducted due to data availability.

**Conclusions:**

The eGFR discordance serves as a meaningful indicator of adverse health outcomes. The lack of a consensus on the cut-off value of eGFR discordance and the mixture use of eGFR equations warrants attention.

KEY LEARNING POINTS **What was known:**

- The intra-individual difference between creatinine-based estimated glomerular filtration rate (eGFR) and cystatin C–based eGFR, i.e. eGFR discordance, has recently been demonstrated to be associated with mortality, cardiovascular outcomes and renal outcomes.
- There was a lack of comprehensive review to assess the pooled effects of eGFR discordance on adult health.

**This study adds:**

- Individuals with lower eGFRcys relative to eGFRcr have significantly higher risks of mortality and cardiovascular events.
- Those with higher eGFRcys relative to eGFRcr exhibit lower risks of these adverse health outcomes.

**Potential impact:**

- Implementing eGFR discordance assessment in clinical practice may enhance risk stratification and guide treatment strategies.
- Emphasizing the need for further research to standardize eGFR discordance definition and explore its implications across diverse populations.

## INTRODUCTION

An accurate estimated glomerular filtration rate (eGFR) is key in the clinical diagnosis and management of chronic kidney disease (CKD). Common equations for estimating GFR are based on serum creatinine and cystatin C levels. Creatinine is widely used but its levels can be influenced by muscle mass, diet and medications [1, 2]. In contrast, cystatin C is less likely affected by common confounders like muscle mass and diet [3], but may be influenced by alternative non-GFR factors including obesity and glucocorticoids [4]. Although the accuracy of eGFR derived from a single biomarker has been debated [5, 6], several studies have highlighted the advantages of using cystatin C–based eGFR (eGFRcys) over creatinine-based eGFR (eGFRcr), as it offers better accuracy in predicting cardiovascular disease (CVD), end-stage kidney disease (ESKD) progression and mortality [7–10].

Since both creatinine and cystatin C are influenced by non-GFR determinants, it is common to identify situations where large discordances exist between eGFRcr and eGFRcys [11]. For example, eGFRcys can be as low as 70% of eGFRcr or >15 mL/min/1.73 m^2^ lower than eGFRcr [12, 13]. Such discordances may lead to differences in diagnosis or staging of CKD and/or eligibility for clinical treatments, and cannot be attributed to kidney function alone.

Various hypotheses have been proposed to explain situations where large discordances exist. Grubb *et al*. (2015) highlighted significantly higher ratios of cystatin C/creatinine, β_2_-microglobulin/creatinine, and beta-trace protein/creatinine in people with eGFRcys ≤60% of eGFRcr, compared with those with eGFRcys within 90%–110% of eGFRcr [14]. Given the similar molecular sizes of cystatin C, β_2_-microglobulin and beta-trace protein, and the fact that their production is not co-regulated, Grubb *et al*. proposed that a reduction in the pore diameter of the glomerular membrane impaired the filtration of these proteins, resulting in the observed ratio elevations [the ‘shrunken pore syndrome’ (SPS)] [13, 14]. Considering that the molecular weight of cystatin C is 13 kDa, while creatinine is merely 113 Da, this presence of eGFR discordance may indicate enhanced selective filtration of medium-sized molecules (5–30 kDa) in the kidneys [12, 15]. Thus, another term, ‘selective glomerular hypofiltration syndrome’ was proposed in early 2023 [15].

Alternatively, eGFR discordance may relate to the differential production of creatinine and cystatin C [4]. The degree of eGFR discordance is purported to be a useful indicator of muscle mass [16]. Individuals with the lowest eGFRcys/eGFRcr ratio have the largest absolute underestimation of mGFR by eGFRcys and overestimation by eGFRcr (particularly in the setting of muscle wasting) [17].

Immediately following Grubb *et al*.’s study in 2015, several researchers reported the association of SPS with an increase in mortality in patients undergoing elective coronary artery bypass grafting [18] and in healthy seniors (aged 60 years and above) [19, 20]. Since 2020, there is an increase in studies showed that eGFR discordance was associated with various adverse outcomes, including kidney disease, CVD and mortality [11, 21, 22]. These associations suggested that eGFR discordance could serve as a potential marker for identifying vulnerable populations, which highlights the importance of understanding this phenomenon in depth.

Despite the growing body of literature on eGFR discordance, there is a lack of systematic reviews and meta-analyses to comprehensively evaluate its association with health outcomes. Therefore, this study aims to conduct a systematic review and meta-analysis to assess the pooled effect of eGFR discordance on mortality, kidney and CVD.

## MATERIALS AND METHODS

This review has been registered on PROSPERO (CRD42024540635). We followed the Preferred Reporting Items for Systematic Reviews and Meta-Analysis (PRISMA) statement [23] and Meta-analysis Of Observational Studies in Epidemiology (MOOSE) [24]. One author (Q.L.) performed systematic literature searches on Embase, PubMed and MEDLINE from database inception to 28 April 2024. If study data were not published in the pre-defined discordance categories, authors were contacted via e-mail twice with an interval of a week to obtain the full text if it was not available online. Observational studies of longitudinal and cross-sectional designs were included. Due to resource constraints, we only included English language manuscripts and did not search for unpublished results.

Studies meeting the following criteria were excluded: (i) studies conducted in populations whose kidney function cannot be reliably evaluated by creatinine-based eGFR, such as patients receiving dialysis [25]; (ii) studies involving people with cancer, AIDS or those under intensive care; and (iii) studies that did not have mortality, cardiovascular or kidney diseases as outcomes. Detailed search terms can be found in the Supplementary data, Table S1.

Two authors (Q.L., P.W.) independently reviewed and selected studies for inclusion, with any conflicts resolved through consultation with two additional authors (C.C.-M. and P.B.M.). To ensure comprehensive coverage, one author (Q.L.) thoroughly examined the references of the chosen articles and assessed their relevance after reading the full texts. Furthermore, this author (Q.L.) conducted manual searches for additional relevant studies.

### Quality assessment

Because all the selected studies were observational studies, their qualities were independently assessed by two reviewers (Q.L., P.W.) using the Risk Of Bias In Non-randomized Studies—of Exposure (ROBINS-E), which was designed for evaluating observational studies [26]. The methodological flaws of a study were categorized as low, some concerns, high or very high based on (i) confounding, (ii) measurement of the exposure, (iii) selection of participants, (iv) post-exposure interventions, (v) missing data, (vi) measurement of the outcome and (vii) selection of the reported results. A cohort study was considered biased if the loss to follow-up was 20% or above [27]. Any disparities in judgment raised between the two reviewers were resolved through discussion with a third reviewer (J.S.L.) as needed.

### Data synthesis

Using a predesigned table, information on each study was extracted from the first author's family name, publication year, study type, study location, characteristics of the study population, eGFR calculation method, kidney characteristics, study exposure, the prevalence of eGFR discordance defined by the study, study outcome and main study findings (i.e. subgroup findings were not presented). If a study calculated eGFR using multiple eGFR equations, then the result of each equation would be extracted separately. The outcomes of interest were predefined as mortality, cardiovascular events and renal events.

In this review, the direction of discordance was arbitrarily defined as the derivation from eGFRcr, which means eGFRcr was the subtrahend or denominator. If a study reported results in the opposite direction, such as eGFRcr being the minuend or numerator, the results would be reversed to ensure all findings were presented in the same direction for ease of comparison. Data were synthesized and compared among studies if they have the same reference group definition and eGFR discordance calculation methods.

Data reported in the median [interquartile range (IQR)] were converted to the mean [standard deviation (SD)] following established methods [28]. One author (F.K.H.) offered advice on methodology and statistical approaches. We used random-effects model with the restricted maximum likelihood method to pool the hazard ratio (HR) or odds ratio (OR) from individual studies [29]. Heterogeneity between studies relative to total variance was examined using the I^2^ statistic [30]. Subgroup meta-analyses was performed subject to data availability. Funnel plots were used to evaluate the risk of biased results [31]. Statistics analyses were performed using STATA 17 (StataCorp, USA) and Robvis (https://mcguinlu.shinyapps.io/robvis/) [32].

### Sensitivity analysis

Leave-one-out analysis was performed to identify influential studies by conducting the meta-analysis multiple times while removing one of the included studies during each iteration. Results are presented as leave-one-out figures.

## RESULTS

### Identification of studies

**Figure 1:**
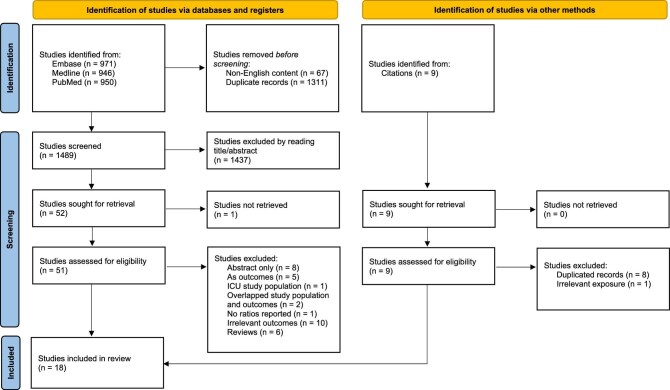
The PRISMA flow diagram.

After removing duplicated studies, 1489 potentially relevant studies were identified. Initial screening based on title and abstracts resulted in 51 studies retrieved for further evaluation. Following full-text assessment, 33 studies were excluded, leaving 18 studies [11, 17, 21, 22, 33–46]. Another nine studies were identified through reading citations, and all were excluded (Fig. 1). All the included studies were either of cohort design or cross-sectional design or covered both.

### Characteristics of the included studies

All selected studies were published between 2020 and 2024. The 18 studies included 20 study populations. Sample sizes ranged from 373 to 363 494 individuals, with a median of 4252 individuals. Nineteen out of 20 populations had a median or mean age of 50 years or older, with the oldest age being 84 years. One study had a much younger population, with a median (IQR) age of 37 (27–48) years [44]. Except for one study where all participants were female, all other studies included both males and females (Tables 1 and 2) [41].

**Table 1:** The study characteristics of the included studies.

				Study population							
	Author	Study	Follow-up					eGFR calculation		Prevalence of exposure,	
ID	year	type	duration	Size	Age	Sex	Country	method	Exposure	as categorical variable *n* (%)	Outcome
								
1	Akesson *et al*. 2020	Cohort	Median, years: 5.6	2781 people from LCS cohort	Median (2.5–97.5 percentiles), years: 63 (20–85)	Male, *n* (%): 1449 (51.4) Female, *n* (%): 1332 (48.6)	Sweden	LMRcr: LMrev equation CAPAcys: CAPA equation CKD-EPIcr, CKD-EPIcys: 2012 CKD-EPI equation mGFR: plasma clearance of iohexol FAScr, FAScys: FAS equation	CAPAcys/LMRcr ratio CKD-EPIcys/CKD-EPIcr ratio FAScys/FAScr ratio	Size (%) CAPAcys/LMRcr ratio <0.70: 645 (23.2) CAPAcys/LMRcr ratio 0.70–0.84: 699 (25.1) CAPAcys/LMRcr ratio 0.85–0.99: 728 (26.2) CAPAcys/LMRcr ratio ≥1.00: 709 (25.5)	1. All-cause mortality 2. Cause-specific mortality by cancer, CVD, diabetes and CKD
2	Carrero *et al*. 2023	Cohort	Median (IQR), years: 4.5 (2.3–6.8)	158 601 people from SCREAM project	Mean (SD), years: 62 (18)	Male, %: 52 Female, %: 48	Sweden	eGFRcr: 2021 CKD-EPI race-free equation eGFRcys: 2012 CKD-EPI equation	Percent eGFRdiff, defined as (eGFRcys – eGFRcr)/eGFRcr Absolute eGFRdiff, defined as eGFRcys – eGFRcr	Not applicable	1. Kidney failure with replacement therapy 2. AKI 3. HF 4. Atherosclerotic CVD 5. All-cause death 6. Cardiovascular death
3	Chen *et al*. 2022a	Cohort	Incident of ESKD Median (IQR), years: 4.7 (2.6–7.5) All-cause mortality Median (IQR), years: 7.2 (4.4–9.7)	4956 people from CRIC study	Mean (SD), years: 59.5 (10.5)	Male, *n* (%): 2800 (56.5) Female, *n* (%): 2156 (43.5)	USA	eGFRcr: 2021 CKD-EPI race-free equation eGFRcys: 2012 CKD-EPI equation	eGFRdiffcys-cr, defined as eGFRcys – eGFRcr	Size (%) eGFRdiffcys-cr <–15 mL/min/1.73 m^2^: 390 (7.9) eGFRdiffcys-cr –15 to 15 mL/min/1.73 m^2^: 3318 (66.9) eGFRdiffcys-cr ≥15 mL/min/1.73 m^2^: 1248 (25.2)	1. Incident ESKD 2. All-cause mortality
4	Chen *et al*. 2022b	Cohort	Median (IQR), years: 3.5 (1.5–7.1)	4512 people from CRIC study, without prevalent HF	Mean (SD), years: 59.4 (10.7)	Male, *n* (%): 2531 (56.1) Female, *n* (%): 1981 (43.9)	USA	eGFRcr: 2021 CKD-EPI race-free equation eGFRcys: 2012 CKD-EPI equation	eGFRdiff, defined as eGFRcys – eGFRcr	Size (%) eGFRdiff <–15 mL/min/1.73 m^2^: 340 (7.5) eGFRdiff –15 to 15 mL/min/1.73 m^2^: 2977 (66.0) eGFRdiff ≥15 mL/min/1.73 m^2^: 1195 (26.5)	Incident HF hospitalization
5	Farrington *et al*. 2023	Cohort	Minimum 25 years	13 197 people from the ARIC study without prevalent kidney failure at visit 2	Mean (SD), years: 57 (6)	Male, *n* (%): 5778 (44) Female, *n* (%): 7419 (56)	USA	eGFRcr: 2021 CKD-EPI race-free equation eGFRcys: 2012 CKD-EPI equation	eGFRcys/eGFRcr ratio	Size (%) eGFRcys >30% lower than eGFRcr: 937 (7) eGFRcys >30% higher than eGFRcr: 343 (3)	1. Incident kidney failure, HF, AKI 2. Death
6	He *et al*. 2024a	Cohort	INDEED Median (IQR), years: 3.83 (3.47–4.18) NHANES Median (IQR), years: 15.17 (7.7–17.58) UK Biobank Median (IQR), years: 13.51 (12.68–14.39)	All with diabetes and without history of CVDs. 8129 people from INDEED 1634 people from NHANES 29 358 people from the UK Biobank	Mean (SD), years: INDEED: 60.7 (10.0) NHANES: 62.5 (14.4) UK Biobank: 59.4 (7.3)	INDEED: Male, *n* (%): 6440 (79.2) Female, *n* (%): 1689 (20.8) NHANES: Male, *n* (%): 877 (53.7) Female, *n* (%): 757 (46.3) UK Biobank: Male, *n* (%): 17 787 (60.6) Female, *n* (%): 11 571 (39.4)	China, USA and UK	eGFRcr: 2021 CKD-EPI race-free equation eGFRcys: 2012 CKD-EPI equation	eGFRabdiff, defined as eGFRcys – eGFRcr, eGFRrediff, defined as eGFRcys/eGFRcr	INDEED Size (%) eGFRabdiff <–15 mL/min/1.73 m^2^: 2750 (33.8) eGFRabdiff –15 to 15 mL/min/1.73 m^2^: 4584 (56.4) eGFRabdiff ≥15 mL/min/1.73 m^2^: 795 (9.8) eGFRrediff ≥0.6: 7802 (96.0) eGFRrediff <0.6: 327 (4.0) NHANES Size (%) eGFRabdiff <–15 mL/min/1.73 m^2^: 211 (12.9)	1. All-cause mortality 2. Incident cardiovascular events, defined as myocardial infarction, HF, atrial fibrillation and stroke
										eGFRabdiff –15 to 15 mL/min/1.73 m^2^: 1104 (67.6) eGFRabdiff ≥15 mL/min/1.73 m^2^: 319 (19.5) eGFRrediff ≥0.6: 1602 (98) eGFRrediff <0.6: 32 (2.0) UK Biobank Size (%) eGFRabdiff <–15 mL/min/1.73 m^2^: 11 563 (39.4) eGFRabdiff –15 to 15 mL/min/1.73 m^2^: 16 994 (57.9) eGFRabdiff ≥15 mL/min/1.73 m^2^: 801 (2.7) eGFRrediff ≥0.6: 28 411 (96.8) eGFRrediff <0.6: 947 (3.2)	
7	He *et al*. 2024b	Cohort	Median (IQR), years: 13.59 (12.77–14.43)	25 825 people with diabetes free of diabetic microvascular complications from the UK Biobank	Mean (SD), years: 59.1 (7.3)	Male, *n* (%): 15 589 (60.4) Female, *n* (%): 10 236 (39.6)	UK	eGFRcr: 2021 CKD-EPI race-free equation eGFRcys: 2012 CKD-EPI equation	eGFRabdiff, defined as eGFRcys – eGFRcr eGFRrediff, defined as eGFRcys/eGFRcr	Size (%) eGFRabdiff <–15: 10 045 (38.9) eGFRabdiff –15 to 15: 15 028 (58.2) eGFRabdiff ≥15: 752 (2.9) eGFRrediff ≥0.6: 25 247 (97.8) eGFRrediff <0.6: 578 (2.2)	Incidence of diabetic kidney disease
8	Heo *et al*. 2024	Cohort	Median (IQR), years: 11.7	363 494 people from the UK Biobank without a prior diagnosis of atrial fibrillation or a history of related procedures, including catheter ablation	Mean (SD), years: 56.2 (8.1)	Male, *n* (%): 172 004 (47.3) Female, *n* (%): 191 490 (52.7)	UK	eGFRcr: 2009 CKD-EPI race-dependent equation, 2021 CKD-EPI race-free equation (sensitivity analysis only) eGFRcys: 2012 CKD-EPI equation	eGFRdiff, defined as eGFRcys – eGFRcr, which was calculated using 2009 CKD-EPI race-dependent equation Race-free eGFRdiff, defined as eGFRcys – eGFRcr, which was calculated using 2021 CKD-EPI race-free equation	Size (%) eGFRdiff <–15 mL/min/1.73 m^2^: 48 899 (13.5) eGFRdiff –15 to 15 mL/min/1.73 m^2^: 276 960 (76.2) eGFRdiff ≥15 mL/min/1.73 m^2^: 37 635 (10.4)	Incident atrial fibrillation
9	Herou *et al*. 2022	Cohort	Median (IQR), years: 6.5 (5.1–8.2)	3993 people underwent CABG, SAVR, or CABG + SAVR	Mean (SD), years: 68 (10)	Male, *n* (%): 3063 (77) Female, *n* (%): 930 (23)	Sweden	eGFRcr: 2012 CKD-EPI race-dependent equation eGFRcys: 2012 CKD-EPI equation eGFR LMrev: LMrev creatinine equation eGFR CAPA: CAPA cystatin C equation	SPS, defined as eGFRcys ≤60% of eGFRcr, and by eGFR CAPA ≤60% of eGFR LMrev separately	Size (%), by CKD-EPI equation eGFRcys ≤60% of eGFRcr: 296 (7.4) Size (%), by CAPA and LMrev equation eGFR CAPA ≤60% of eGFR LMrev: 92 (2.3)	10-year mortality
10	Jonsson *et al*. 2021	Cohort	Not available	996 people with hip fracture.	Median (IQR), years: 84 (77–90)	Male, %: 29 Female, %:71	Sweden	eGFRcr: LMrev creatinine equation eGFRcys: CAPA cystatin C equation	SPS, defined as eGFRcys/eGFRcr ≤0.7	Size (%) eGFRcys/eGFRcr ≤0.7: 87 (8.7)	1. All-cause 1-year mortality 2. All-cause 90-day mortality
								
11	Kim *et al*. 2021	Cohort	Median, years: 4.1	2076 people with CKD from KNOW-CKD study	Mean (SD), years: 53.5 (12.2)	Male, *n* (%): 1265 (60.9) Female, *n* (%): 811 (39.1)	Korea	2012 CKD-EPI equation	eGFRdiff, defined as eGFRcreat – eGFRcys	Size (%) eGFRdiff <–2.3 mL/min/1.73 m^2^: 691 (33.3) –2.3 ≤ eGFRdiff < 4.0 mL/min/1.73 m^2^: 690 (33.3) eGFRdiff ≥4.0 mL/min/1.73 m^2^: 695 (33.4)	Newly occurred MACE, defined as death from cardiovascular causes and any non-fatal cardiovascular events that required hospitalization
12	Ljungberg *et al*. 2019	Case–control	Not applicable	Case: 336 survey participants who received surgery for aortic stenosis. Control: 671 people matched for sex, age, type and date of survey, and geographical area	Mean (95% CI), year At survey Case: 56.7 (55.8, 57.6) Control: 56.7 (56.0, 57.3)	Female (95% CI), % Case: 48 (43, 53) Control: 48 (44, 52)	Sweden	eGFRcr: LMrev creatinine equation eGFRcys: CAPA cystatin C equation	eGFRcys/eGFRcr Z (ln) ratio, defined as Z-score of ln(eGFRcys/eGFRcr)	Not applicable.	Aortic valve replacement due to aortic stenosis
13	Malmgren *et al*. 2022	Cohort	Minimum 10 years	849 females from OPRA cohort, without sarcopenia or taking glucocorticoids.	Mean (SD), years: 75.2 (0.14)	Female, *n* (%): 849 (100)	Sweden	eGFRcr: 2012 CKD-EPI race-dependent equation, LMrev creatinine equation (sensitivity analysis only) eGFRcysC: 2012 CKD-EPI equation, CAPA cystatin C equation (sensitivity analysis only)	eGFRcysC/eGFRcr <0.6	Size (%), by CKD-EPI equation eGFRcysC/eGFRcr ratio <0.6: 80 (9.4) eGFRcysC/eGFRcr ratio 0.6–0.69: 85 (10.0) eGFRcysC/eGFRcr ratio 0.7–0.79: 140 (16.5) eGFRcysC/eGFRcr ratio 0.8–0.89: 178 (21.0) eGFRcysC/eGFRcr ratio ≥0.9: 366 (43.1) Size (%), by CAPA and LMrev equation eGFRcysC/eGFRcr ratio <0.6: 22 (2.6) eGFRcysC/eGFRcr ratio 0.6–0.69: 39 (4.6) eGFRcysC/eGFRcr ratio 0.7–0.79: 56 (6.6) eGFRcysC/eGFRcr ratio 0.8–0.89: 99 (11.7) eGFRcysC/eGFRcr ratio ≥0.9: 633 (74.5)	10-year mortality
14	Potok *et al*. 2020	Cohort	Not available	9092 people from SPRINT study.	Mean (SD), years: 68 (9)	Male, *n* (%): 5842 (64) Female, *n* (%): 3250 (36)	USA	2012 CKD-EPI equation	eGFRDiff, defined as eGFRcys – eGFRcr	Size (%) eGFRDiff <–15: 1230 (14) eGFRDiff -15 to 15: 6471 (71) eGFRDiff ≥15: 1391 (15)	1. Cardiovascular events, including myocardial infarction, stroke, acute coronary syndrome and HF 2. Total mortality
								
15	Söderström *et al*. 2021	Nested case–control	Not applicable	Case: 545 survey participants who experienced first-ever myocardial infarction Control: 1054 people matched for sex, age, time and type of survey, and geographical area	Mean, year Male Case: 53.6 Control: 53.4 Female Case: 58.3 Control: 58.1	Male, *n* (%) Case: 387 (71.0) Control: 752 (71.3)	Sweden	eGFRcr: LMrev creatinine equation eGFRcys: CAPA cystatin C equation	eGFRcys/eGFRcr Z (ln) ratio, defined as Z-score of ln(eGFRcys/eGFRcr)	Not applicable	First ever myocardial infarction
13	Wu *et al*. 2022	Cohort	Mean, months: 27.7	536 people with IgA nephropahty or membranous nephropathy	Median (IQR), years: 37 (27–48)	Male, *n* (%): 262 (49) Female, *n* (%): 274 (51)	China	2012 CKD-EPI equation	SPS, defined as eGFRcys <70% eGFRcr	Size (%) eGFRcys <70% eGFRcr: 44 (8.2)	1. ESKD, defined as eGFRcr ≤15 mL/min/1.73 m^2^ or the initation of renal placement therapy 2. Severe eGFR decline, defined as stable serum creatinine doubling or ≥50% eGFRcr decline compared with its baseline value
14	Xhakollari *et al*. 2021	Cohort	Median, years: 1.8	373 people hospitalized for newly diagnosed or exacerbated acute HF from HARVEST-Malmo Study	Mean (SD), years: 74.8 (12.1)	Male, *n* (%): 255 (68.4) Female, *n* (%): 118 (31.6)	Sweden	2012 CKD-EPI equation	SPS, defined as eGFRcys ≤60% of eGFRcr	Size (%) eGFRcys/eGFRcr ratio ≤0.6: 94 (25.2) eGFRcys/eGFRcr ratio >0.6: 279 (74.8)	1. All-cause mortality 2. 30-day rehospitalization due to cardiac causes
15	Zhang *et al*. 2023	Cohort, Cross-sectional	Median, years: 2.8	5050 people underwent elective PCI	Median (IQR), years Non-SPS group: 66 (58–73) SPS group: 69 (61–76)	Non-SPS group Male, *n* (%): 3472 (78.9) Female, *n* (%): 929 (21.1) SPS group Male, *n* (%): 487 (75.0) Female, *n* (%): 162 (25.0)	China	eGFRcr: 2021 CKD-EPI race-free equation eGFRcys: 2012 CKD-EPI equation	SPS, defined as eGFRcys <60% eGFRcr, and eGFRcys <70% eGFRcr, respectively	Size (%) eGFRcys <60% eGFRcr: 649 (12.9)	1. CA-AKI, defined as a relative increase in SCr ≥50% or an absolute SCr increase ≥0.3 mg/dL within 48 h after contrast medium exposure 2. Mortality

CA-AKI, contrast-associated AKI; CABG, coronary artery bypass grafting; PCI, percutaneous coronary intervention; SAVR, surgical aortic valve replacement.

**Table 2:** Renal characteristics and the main findings of the included studies.

ID	Author year	Study type	Renal characteristics	Main findings
1	Akesson *et al*. 2020	Cohort	Median (2.5–97.5 percentiles): CAPAcys, mL/min/1.73 m^2^: 47 (10–107) LMRcr, mL/min/1.73 m^2^: 61 (11–111) CKD-EPIcr, mL/min/1.73 m^2^: 66 (11–127) CKD-EPIcys, mL/min/1.73 m^2^: 46 (10–112) FAScr, mL/min/1.73 m^2^: 62 (13–140) FAScys, mL/min/1.73 m^2^: 48 (15–109) mGFR, mL/min/1.73 m^2^: 57 (10–118) CAPAcys/LMRcr ratio: 0.86 (0.46–1.29) CKD-EPIcys/CKD-EPIcr ratio: 0.78 (0.41–1.26) FAScys/FAScr ratio: 0.84 (0.47–1.34)	1. All-cause mortality, HR (95% CI) CAPAcys/LMRcr ratio <0.60: 3.3 (2.5, 4.5) CAPAcys/LMRcr ratio <0.70: 3.0 (2.4, 3.7) CAPAcys/LMRcr ratio 0.70–0.84: 2.2 (1.8, 2.7) CAPAcys/LMRcr ratio 0.85–0.99: 1.3 (1.1, 1.6) CAPAcys/LMRcr ratio ≥1.00: Reference CKD-EPIcys/CKD-EPIcr ratio <0.70: 2.4 (1.9, 3.1) CKD-EPIcys/CKD-EPIcr ratio 0.70–0.84: 1.5 (1.2, 1.9) CKD-EPIcys/CKD-EPIcr ratio 0.85–0.99: 1.2 (0.9, 1.6) CKD-EPIcys/CKD-EPIcr ratio ≥1.00: Reference FAScys/FAScr ratio <0.70: 2.7 (2.2, 3.5) FAScys/FAScr ratio 0.70–0.84: 1.9 (1.5, 2.3) FAScys/FAScr ratio 0.85–0.99: 1.2 (1.0, 1.5) FAScys/FAScr ratio ≥1.00: Reference 2. Cancer mortality, HR (95% CI) CAPAcys/LMRcr ratio <0.70: 3.3 (2.3, 4.9) CAPAcys/LMRcr ratio 0.70–0.84: 2.4 (1.7, 3.3) CAPAcys/LMRcr ratio 0.85–0.99: 1.3 (1.0, 1.8) CAPAcys/LMRcr ratio ≥1.00: Reference 3. CVD mortality, HR (95% CI) CAPAcys/LMRcr ratio <0.70: 2.4 (1.6, 3.7) CAPAcys/LMRcr ratio 0.70–0.84: 1.7 (1.1, 2.5) CAPAcys/LMRcr ratio 0.85–0.99: 1.2 (0.7, 1.8) CAPAcys/LMRcr ratio ≥1.00: Reference 4. Diabetes mortality, HR (95% CI) CAPAcys/LMRcr ratio <0.70: 2.0 (0.8, 4.7) CAPAcys/LMRcr ratio 0.70–0.84: 1.9 (0.8, 4.7) CAPAcys/LMRcr ratio 0.85–0.99: 1.2 (0.5, 3.3) CAPAcys/LMRcr ratio ≥1.00: Reference 5. CKD mortality, HR (95% CI) CAPAcys/LMRcr ratio <0.70: 5.1 (0.6, 46.0) CAPAcys/LMRcr ratio 0.70–0.84: 3.3 (0.3, 33.7) CAPAcys/LMRcr ratio 0.85–0.99: 2.2 (0.2, 25.6) CAPAcys/LMRcr ratio ≥1.00: Reference
2	Carrero *et al*. 2023	Cohort	Mean (SD) eGFRcr, mL/min/1.73 m^2^: 80 (26) eGFRcys, mL/min/1.73 m^2^: 73 (31) Median (IQR) %eGFRdiff, –10 (–27 to 6)	1. Kidney failure with replacement therapy Quartiles of Percent eGFRdiff, HR (95% CI) Quartile 1 (eGFRcys << eGFRcr): 1.36 (1.17, 1.58) Quartile 2 (eGFRcys < eGFRcr): 1.08 (0.94, 1.25) Quartile 3 (eGFRcys ≈ eGFRcr): Reference Quartile 4 (eGFRcys > eGFRcr): 0.79 (0.69, 0.92) Quartiles of absolute eGFRdiff, HR (95% CI) Quartile 1 (eGFRcys<<eGFRcr): 2.46 (1.98, 3.05) Quartile 2 (eGFRcys < eGFRcr): 1.20 (1.05, 1.36) Quartile 3 (eGFRcys ≈ eGFRcr): Reference Quartile 4 (eGFRcys > eGFRcr): 0.57 (0.48, 0.69) 2. AKI Quartiles of Percent eGFRdiff, HR (95% CI) Quartile 1 (eGFRcys << eGFRcr): 2.62 (2.42, 2.85) Quartile 2 (eGFRcys < eGFRcr): 1.53 (1.40, 1.67) Quartile 3 (eGFRcys ≈ eGFRcr): Reference Quartile 4 (eGFRcys > eGFRcr): 0.67 (0.59, 0.75) Quartiles of absolute eGFRdiff, HR (95% CI) Quartile 1 (eGFRcys << eGFRcr): 3.10 (2.85, 3.36) Quartile 2 (eGFRcys < eGFRcr): 1.60 (1.48, 1.72) Quartile 3 (eGFRcys ≈ eGFRcr): Reference Quartile 4 (eGFRcys > eGFRcr): 0.64 (0.57, 0.72)
				3. Atherosclerotic CVD Quartiles of Percent eGFRdiff, HR (95% CI) Quartile 1 (eGFRcys << eGFRcr): 1.42 (1.33, 1.51) Quartile 2 (eGFRcys < eGFRcr): 1.19 (1.11, 1.27) Quartile 3 (eGFRcys ≈ eGFRcr): Reference Quartile 4 (eGFRcys > eGFRcr): 0.79 (0.73, 0.86) Quartiles of absolute eGFRdiff, HR (95% CI) Quartile 1 (eGFRcys << eGFRcr): 1.46 (1.37, 1.56)
				Quartile 2 (eGFRcys < eGFRcr): 1.19 (1.12, 1.26) Quartile 3 (eGFRcys ≈ eGFRcr): Reference Quartile 4 (eGFRcys > eGFRcr): 0.78 (0.72, 0.85) 4. HF Quartiles of Percent eGFRdiff, HR (95% CI) Quartile 1 (eGFRcys << eGFRcr): 2.04 (1.92, 2.17) Quartile 2 (eGFRcys < eGFRcr): 1.33 (1.25, 1.41) Quartile 3 (eGFRcys ≈ eGFRcr): Reference Quartile 4 (eGFRcys > eGFRcr): 0.76 (0.70, 0.83) Quartiles of absolute eGFRdiff, HR (95% CI) Quartile 1 (eGFRcys << eGFRcr): 2.20 (2.07, 2.34) Quartile 2 (eGFRcys < eGFRcr): 1.41 (1.34, 1.49) Quartile 3 (eGFRcys ≈ eGFRcr): Reference Quartile 4 (eGFRcys > eGFRcr): 0.73 (0.67, 0.80) 5. Cardiovascular death Quartiles of Percent eGFRdiff, HR (95% CI) Quartile 1 (eGFRcys << eGFRcr): 2.48 (2.32, 2.66) Quartile 2 (eGFRcys < eGFRcr): 1.40 (1.30, 1.50) Quartile 3 (eGFRcys ≈ eGFRcr): Reference Quartile 4 (eGFRcys > eGFRcr): 0.85 (0.77, 0.94) Quartiles of absolute eGFRdiff, HR (95% CI) Quartile 1 (eGFRcys << eGFRcr): 2.87 (2.69, 3.06) Quartile 2 (eGFRcys < eGFRcr): 1.50 (1.41, 1.58) Quartile 3 (eGFRcys ≈ eGFRcr): Reference Quartile 4 (eGFRcys > eGFRcr): 0.78 (0.70, 0.86) 6. All-cause death Quartiles of Percent eGFRdiff, HR (95% CI) Quartile 1 (eGFRcys << eGFRcr): 2.62 (2.54, 2.72) Quartile 2 (eGFRcys < eGFRcr): 1.46 (1.41, 1.52) Quartile 3 (eGFRcys ≈ eGFRcr): Reference Quartile 4 (eGFRcys > eGFRcr): 0.80 (0.77, 0.84) Quartiles of absolute eGFRdiff, HR (95% CI) Quartile 1 (eGFRcys << eGFRcr): 2.88 (2.79, 2.98) Quartile 2 (eGFRcys < eGFRcr): 1.49 (1.45, 1.54) Quartile 3 (eGFRcys ≈ eGFRcr): Reference Quartile 4 (eGFRcys > eGFRcr): 0.74 (0.70, 0.77)
3	Chen *et al*. 2022a	Cohort	Mean (SD) eGFRcr, mL/min/1.73 m^2^: 49 (16) eGFRcys, mL/min/1.73 m^2^: 54 (23) eGFRdiffcys-cr, mL/min/1.73 m^2^: 6 (16)	1. Incident ESKD Baseline measures, sHR (95% CI) eGFRdiffcys-cr <–15 mL/min/1.73 m^2^: 1.00 (0.65, 1.52) eGFRdiffcys-cr –15 to 15 mL/min/1.73 m^2^: Reference eGFRdiffcys-cr ≥15 mL/min/1.73 m^2^: 0.73 (0.59, 0.89) Time-updated measures, sHR (95% CI) eGFRdiffcys-cr <–15 mL/min/1.73 m^2^: 1.83 (1.10, 3.04) eGFRdiffcys-cr –15 to 15 mL/min/1.73 m^2^: Reference eGFRdiffcys-cr ≥15 mL/min/1.73 m^2^: 0.50 (0.35, 0.71) 2. All-cause mortality Baseline measures, HR (95% CI) eGFRdiffcys-cr <–15 mL/min/1.73 m^2^: 1.86 (1.40, 2.48) eGFRdiffcys-cr –15 to 15 mL/min/1.73 m^2^: Reference eGFRdiffcys-cr ≥15 mL/min/1.73 m^2^: 0.68 (0.58, 0.81)
				Time-updated measures, HR (95% CI) eGFRdiffcys-cr <–15 mL/min/1.73 m^2^: 3.03 (2.19, 4.19) eGFRdiffcys-cr –15 to 15 mL/min/1.73 m^2^: Reference eGFRdiffcys-cr ≥15 mL/min/1.73 m^2^: 0.58 (0.45, 0.75)
4	Chen *et al*. 2022b	Cohort	Mean (SD) eGFRcr, mL/min/1.73 m^2^: 49 (16) eGFRcys, mL/min/1.73 m^2^: 55 (23) eGFRdiff overall, mL/min/1.73 m^2^: 6 (16) eGFRdiff <–15 mL/min/1.73 m^2^: –24 (8) eGFRdiff -–15 to 15 mL/min/1.73 m^2^: 2 (7) eGFRdiff ≥15 mL/min/1.73 m^2^: 26 (10)	Incident HF hospitalization Baseline measures, sHR (95% CI) eGFRdiff, per –15 mL/min/1.73 m^2^: 1.20 (1.07, 1.34) eGFRdiff <–15 mL/min/1.73 m^2^: 1.14 (0.77, 1.70) eGFRdiff –15 to 15 mL/min/1.73 m^2^: Reference eGFRdiff ≥15 mL/min/1.73 m^2^: 0.78 (0.61, 1.01) Time-updated measures, sHR (95% CI) eGFRdiff, per –15 mL/min/1.73 m^2^: 1.36 (1.18, 1.55)
				eGFRdiff <–15 mL/min/1.73 m^2^: 1.99 (1.39, 2.86) eGFRdiff –15 to 15 mL/min/1.73 m^2^: Reference eGFRdiff ≥15 mL/min/1.73 m^2^: 0.67 (0.49, 0.91)
5	Farrington *et al*. 2023	Cohort	Mean (SD) eGFRcr, mL/min/1.73 m^2^: 97 (14) eGFRcys, mL/min/1.73 m^2^: 91 (19) Percent difference between eGFRcr and eGFRcys: 5 (17) Median (IQR) Percent difference between eGFRcr and eGFRcys: 4 (–5, 16)	1. Kidney failure, HR (95% CI) eGFRcys >30% lower than eGFRcr: 1.53 (1.07, 2.18) 0.7 ≤ eGFRcys/eGFRcr ≤ 1.3: Reference eGFRcys >30% higher than eGFRcr: 0.62 (0.36, 1.08) 2. HF, HR (95% CI) eGFRcys >30% lower than eGFRcr: 1.58 (1.39, 1.80) 0.7 ≤ eGFRcys/eGFRcr ≤ 1.3: Reference eGFRcys >30% higher than eGFRcr: 0.90 (0.69, 1.17) 3. AKI, HR (95% CI) eGFRcys >30% lower than eGFRcr: 1.76 (1.52, 2.03) 0.7 ≤ eGFRcys/eGFRcr ≤ 1.3: Reference eGFRcys >30% higher than eGFRcr: 0.65 (0.48, 0.87) 4. Death, HR (95% CI) eGFRcys >30% lower than eGFRcr: 1.61 (1.47, 1.76) 0.7 ≤ eGFRcys/eGFRcr ≤ 1.3: Reference eGFRcys >30% higher than eGFRcr: 0.76 (0.63, 0.92)
6	He *et al*. 2024a	Cohort	Mean (SD) INDEED eGFRcr, mL/min/1.73 m^2^: 95.54 (14.70) eGFRcys, mL/min/1.73 m^2^: 88.73 (22.53) eGFRabdiff, mL/min/1.73 m^2^: –6.81 (18.17) eGFRrediff, ratio: 0.93 (0.20) NHANES eGFRcr, mL/min/1.73 m^2^: 84.78 (25.85) eGFRcys, mL/min/1.73 m^2^: 87.15 (28.78) eGFRabdiff, mL/min/1.73 m^2^: 2.38 (16.33) eGFRrediff, ratio: 1.04 (0.22) UK Biobank eGFRcr, mL/min/1.73 m^2^: 92.79 (16.35) eGFRcys, mL/min/1.73 m^2^: 81.39 (19.46) eGFRabdiff, mL/min/1.73 m^2^: –11.40 (14.03) eGFRrediff, ratio: 0.80 (0.16)	1. All-cause mortality INDEED, HR (95% CI) eGFRabdiff, per +1 SD: 0.77 (0.69, 0.86) eGFRabdiff <–15 mL/min/1.73 m^2^: 1.38 (1.12, 1.70) eGFRabdiff –15 to 15 mL/min/1.73 m^2^: Reference eGFRabdiff ≥15 mL/min/1.73 m^2^: 0.76 (0.49, 1.19) eGFRrediff, per +10%: 0.88 (0.84, 0.93) eGFRrediff ≥0.6: Reference eGFRrediff <0.6: 1.89 (1.41, 2.53) NHANES, HR (95% CI) eGFRabdiff, per +1 SD: 0.70 (0.65, 0.76) eGFRabdiff <–15 mL/min/1.73 m^2^: 1.43 (1.18, 1.73) eGFRabdiff –15 to 15 mL/min/1.73 m^2^: Reference eGFRabdiff ≥15 mL/min/1.73 m^2^: 0.55 (0.45, 0.67) eGFRrediff, per +10%: 0.87 (0.84, 0.90) eGFRrediff ≥0.6: Reference eGFRrediff <0.6: 2.45 (1.66, 3.62) UK Biobank, HR (95% CI) eGFRabdiff, per +1 SD: 0.66 (0.65, 0.68) eGFRabdiff <–15 mL/min/1.73 m^2^: 1.66 (1.57, 1.75) eGFRabdiff –15 to 15 mL/min/1.73 m^2^: Reference eGFRabdiff ≥15 mL/min/1.73 m^2^: 0.53 (0.42, 0.66) eGFRrediff, per +10%: 0.79 (0.78, 0.80) eGFRrediff ≥0.6: Reference eGFRrediff <0.6: 2.58 (2.35, 2.84) 2. Incident cardiovascular events INDEED, HR (95% CI) eGFRabdiff, per +1 SD: 0.82 (0.74, 0.90) eGFRabdiff <–15 mL/min/1.73 m^2^: 1.31 (1.08, 1.59)
				eGFRabdiff –15 to 15 mL/min/1.73 m^2^: Reference eGFRabdiff ≥15 mL/min/1.73 m^2^: 0.67 (0.44, 1.02) eGFRrediff, per +10%: 0.90 (0.86, 0.95) eGFRrediff ≥0.6: Reference eGFRrediff <0.6: 1.55 (1.10, 2.17) NHANES, HR (95% CI) eGFRabdiff, per +1 SD: 0.68 (0.57, 0.82) eGFRabdiff <–15 mL/min/1.73 m^2^: 0.96 (0.60, 1.55) eGFRabdiff –15 to 15 mL/min/1.73 m^2^: Reference eGFRabdiff ≥15 mL/min/1.73 m^2^: 0.37 (0.22, 0.61) eGFRrediff, per +10%: 0.84 (0.78, 0.91) eGFRrediff ≥0.6: Reference eGFRrediff <0.6: 0.50 (0.12, 2.14) UK Biobank, HR (95% CI) eGFRabdiff, per +1 SD: 0.78 (0.76, 0.81)
				eGFRabdiff <–15 mL/min/1.73 m^2^: 1.37 (1.29, 1.45) eGFRabdiff –15 to 15 mL/min/1.73 m^2^: Reference eGFRabdiff ≥15 mL/min/1.73 m^2^: 0.71 (0.58, 0.87) eGFRrediff, per +10%: 0.87 (0.85, 0.89) eGFRrediff ≥0.6: Reference eGFRrediff <0.6: 1.42 (1.23, 1.63)
7	He *et al*. 2024b	Cohort	Mean (SD) eGFRcr, mL/min/1.73 m^2^: 95.6 (12.6) eGFRcys, mL/min/1.73 m^2^: 84.4 (16.5) eGFRabdiff, mL/min/1.73 m^2^: –11.1 (14.1) eGFRrediff, ratio: 0.9 (0.2)	Diabetic kidney disease HR (95% CI) eGFRabdiff, per –1 SD: 1.56 (1.50, 1.63) eGFRabdiff <–15 mL/min/1.73 m^2^: 1.63 (1.50, 1.76) eGFRabdiff –15 to 15 mL/min/1.73 m^2^: Reference eGFRabdiff ≥15 mL/min/1.73 m^2^: 0.39 (0.31, 0.50) eGFRrediff, per –10%: 1.29 (1.26, 1.33) eGFRrediff ≥0.6: Reference eGFRrediff <0.6: 2.32 (1.94, 2.79) sHR (95% CI) eGFRabdiff, per –1 SD: 1.47 (1.41, 1.54) eGFRabdiff <–15 mL/min/1.73 m^2^: 1.52 (1.41, 1.65) eGFRabdiff –15 to 15 mL/min/1.73 m^2^: Reference eGFRabdiff ≥15 mL/min/1.73 m^2^: 0.39 (0.30, 0.50) eGFRrediff, per –10%: 1.26 (1.22, 1.29) eGFRrediff ≥0.6: Reference eGFRrediff <0.6: 1.84 (1.52, 2.23)
8	Heo *et al*. 2024	Cohort	Mean (SD) eGFRcr, mL/min/1.73 m^2^: 90.7 (13.2) eGFRcys, mL/min/1.73 m^2^: 91 (16.0) eGFRdiff overall, mL/min/1.73 m^2^: –0.6 (13.2) eGFRdiff <–15 mL/min/1.73 m^2^: –22.1 (6.4) eGFRdiff –15 to 15 mL/min/1.73 m^2^: 0.1 (7.8) eGFRdiff ≥15 mL/min/1.73 m^2^: 22.2 (6.9)	Incident atrial fibrillation sHR (95% CI) eGFRdiff, per +10 mL/min/1.73 m^2^: 0.90 (0.88, 0.91) eGFRdiff <–15 mL/min/1.73 m^2^: 1.25 (1.20, 1.30) eGFRdiff –15 to 15 mL/min/1.73 m^2^: Reference eGFRdiff ≥15 mL/min/1.73 m^2^: 0.81 (0.77, 0.87) Race-free eGFRdiff, per +10 mL/min/1.73 m^2^: 0.90 (0.89, 0.91) Race-free eGFRdiff <–15 mL/min/1.73 m^2^: 1.23 (1.19, 1.27) Race-free eGFRdiff –15 to 15 mL/min/1.73 m^2^: Reference Race-free eGFRdiff ≥15 mL/min/1.73 m^2^: 0.88 (0.79, 0.93)
9	Herou *et al*. 2022	Cohort	Mean (SD) eGFRcr, mL/min/1.73 m^2^: 75 (20) eGFRCys, mL/min/1.73 m^2^: 64 (22)	10-year mortality, HR (95% CI) By CKD-EPI equation eGFRcys ≤60% of eGFRcr: 1.96 (1.63, 2.36) eGFRcys >60% of eGFRcr: Reference By CAPA and LMrev equation eGFR CAPA ≤60% of eGFR LMrev: 1.66 (1.25, 2.21) eGFR CAPA >60% of eGFR LMrev: Reference
10	Jonsson *et al*. 2021	Cohort	Percentage eGFRcr ≥90 mL/min/1.73 m^2^: 1% eGFRcr 60–89 mL/min/1.73 m^2^: 16% eGFRcr 30–59 mL/min/1.73 m^2^: 45% eGFRcr 15–29 mL/min/1.73 m^2^: 35% eGFRcr <15 mL/min/1.73 m^2^: 3%	1. All-cause 1-year mortality, HR (95% CI) eGFRcys/eGFRcr ≤0.7: 1.661 (1.155, 2.391) eGFRcys/eGFRcr >0.7: Reference 2. All-cause 90-day mortality, HR (95% CI) eGFRcys/eGFRcr ≤0.7: 1.832 (1.095, 3.063) eGFRcys/eGFRcr >0.7: Reference
11	Kim *et al*. 2021	Cohort	Mean (SD) eGFRcreat, mL/min/1.73 m^2^: 53.4 (30.9) eGFRcys, mL/min/1.73 m^2^: 52.8 (32.5) eGFRdiff, mL/min/1.73 m^2^: 0.6 (11.0)	1. MACE, cause-specific HR (95% CI) eGFRdiff <–2.3 mL/min/1.73 m^2^: Reference –2.3 ≤ eGFRdiff < 4.0 mL/min/1.73 m^2^: 1.74 (1.03, 2.93) eGFRdiff ≥4.0 mL/min/1.73 m^2^: 2.12 (1.26, 3.50) eGFRdiff, per +1 mL/min/1.73 m^2^: 1.03 (1.01, 1.05) 2. Fatal and non-fatal MI and unstable angina, cause-specific HR (95% CI) eGFRdiff, per +1 mL/min/1.73 m^2^: 1.01 (0.98, 1.05) 3. Stroke, cause-specific HR (95% CI) eGFRdiff, per +1 mL/min/1.73 m^2^: 1.03 (0.99, 1.07) 4. Congestive HF, cause-specific HR (95% CI) eGFRdiff, per +1 mL/min/1.73 m^2^: 1.07 (0.99, 1.14) 5. Symptomatic arrhythmia, cause-specific HR (95% CI) eGFRdiff, per +1 mL/min/1.73 m^2^: 1.04 (1.00, 1.08)
12	Ljungberg *et al*. 2019	Case–control	Mean (95% CI) Case eGFRcr, mL/min/1.73 m^2^: 80.0 (78.6, 81.5) eGFRcys, mL/min/1.73 m^2^: 88.9 (86.6, 91.4) eGFRcys/eGFRcr: 1.11 (1.09, 1.13) Control eGFRcr, mL/min/1.73 m^2^: 79.7 (78.8, 80.6) eGFRcys, mL/min/1.73 m^2^: 91.0 (89.6, 92.4) eGFRcys/eGFRcr: 1.14 (1.13, 1.16)	Aortic valve replacement, OR (95% CI) Quartiles of eGFRcys/eGFRcr ratio Quartile 1 (the lowest): 1.00 Quartile 2: 0.91 (0.61, 1.34) Quartile 3: 0.75 (0.51, 1.10) Quartile 4 (the highest): 0.62 (0.40, 0.95) Z (ln) ratio, per +1 SD, OR (95% CI): 0.80 (0.68, 0.95)
13	Malmgren *et al*. 2022	Cohort	Mean (SD), by CKD-EPI equation eGFRcr, mL/min/1.73 m^2^: 75.3 (12.5) eGFRcysC, mL/min/1.73 m^2^: 64.8 (17.1) eGFRcysC/eGFRcr ratio: 0.86 (0.19)	10-year mortality, HR (95% CI) By CKD-EPI equation eGFRcysC/eGFRcr ratio <0.6: 1.6 (1.1, 2.5) eGFRcysC/eGFRcr ratio 0.6–0.69: 1.1 (0.7, 1.8) eGFRcysC/eGFRcr ratio 0.7–0.79: 0.9 (0.6, 1.4) eGFRcysC/eGFRcr ratio 0.8–0.89: 1.0 (0.7, 1.5) eGFRcysC/eGFRcr ratio ≥0.9: Reference By CAPA and LMrev equation eGFRcysC/eGFRcr ratio <0.6: 2.5 (1.4, 4.5) eGFRcysC/eGFRcr ratio 0.6–0.69: 1.3 (0.7, 2.3) eGFRcysC/eGFRcr ratio 0.7–0.79: 1.4 (0.9, 2.3) eGFRcysC/eGFRcr ratio 0.8–0.89: 1.0 (0.7, 1.6) eGFRcysC/eGFRcr ratio ≥0.9: Reference
14	Potok *et al*. 2020	Cohort	Mean (SD) eGFRcr, mL/min/1.73 m^2^: 72 (20) eGFRcys, mL/min/1.73 m^2^: 73 (23) eGFRcr-cys, mL/min/1.73 m^2^: 73 (21) eGFRDiff, mL/min/1.73 m^2^: 0.5 (15)	1. Cardiovascular events, HR (95% CI) eGFRdiff, per +1 SD: 0.89 (0.81, 0.97) 2. Total mortality, HR (95% CI) eGFRdiff, per +1 SD: 0.71 (0.63, 0.82)
15	Söderström *et al*. 2021	Nested case–control	Mean (SD) Male Case eGFRcr, mL/min/1.73 m^2^: 92.1 (12.9) eGFRcys, mL/min/1.73 m^2^: 83.6 (19.8) eGFRcys/eGFRcr ratio: 0.91 (0.20) Male Control eGFRcr, mL/min/1.73 m^2^: 91.2 (10.8) Female Case eGFRcr, mL/min/1.73 m^2^: 86.3 (14.2) eGFRcys, mL/min/1.73 m^2^: 74.8 (18.2) eGFRcys/eGFRcr ratio: 0.87 (0.17) Female Control eGFRcr, mL/min/1.73 m^2^: 97.4 (11.0) eGFRcys, mL/min/1.73 m^2^: 80.7 (16.35) eGFRcys/eGFRcr ratio: 0.93 (0.17) eGFRcys, mL/min/1.73 m^2^: 84.8 (20.5) eGFRcys/eGFRcr ratio: 0.93 (0.19)	First-ever myocardial infarction, OR (95% CI) Quartiles of eGFRcys/eGFRcr Quartile 1 (the lowest): 1.00 Quartile 2: 0.94 (0.70, 1.26) Quartile 3: 0.59 (0.43, 0.80) Quartile 4 (the highest): 0.62 (0.45, 0.85) Z (ln) ratio, per +1 SD, OR (95% CI): 0.92 (0.77, 1.09)
13	Wu *et al*. 2022	Cohort	Median (IQR): eGFRcr, mL/min/1.73 m^2^: 95 (69–112) eGFRcys, mL/min/1.73 m^2^: 76 (58–100) eGFRcys/eGFRcr ratio: 0.85 (0.74–0.96)	Severe eGFR decline, HR (95% CI) eGFRcys <70% eGFRcr: 1.87 (0.86, 4.06) eGFRcys ≥70% eGFRcr: Reference eGFRcys/eGFRcr ratio, per +1 unit: 0.42 (0.06, 2.96)
14	Xhakollari *et al*. 2021	Cohort	Mean (SD) eGFRcr, mL/min/1.73 m^2^: 51.6 (22.6) eGFRcys, mL/min/1.73 m^2^: 37.8 (17.1)	1. All-cause mortality, HR (95% CI) eGFRcys/eGFRcr ratio ≤0.6: 1.99 (1.23, 3.21) 2. 30 day rehospitalization, HR (95% CI) eGFRcys/eGFRcr ratio ≤0.6: 1.82 (1.04, 3.18)
15	Zhang *et al*. 2023	Cohort, cross-sectional	Not available	1. CA-AKI, OR (95% CI) eGFRcys <60% eGFRcr: 4.17 (3.17, 5.46) eGFRcys ≥60% eGFRcr: Reference eGFRcys <70% eGFRcr: 3.58 (2.78, 4.62) eGFRcys ≥70% eGFRcr: Reference 2. Mortality, HR (95% CI) eGFRcys <60% eGFRcr: 1.37 (1.08, 1.74) eGFRcys ≥60% eGFRcr: Reference eGFRcys <70% eGFRcr: 1.27 (1.03, 1.57) eGFRcys ≥70% eGFRcr: Reference

CA-AKI, contrast-associated AKI; MI, myocardial infarction.

The study populations mainly came from the USA and Sweden, followed by the UK and China, with one study involving a Korean population [36]. Most studies focused on the effect of eGFR discordance in patients with kidney diseases or diabetes from the Chronic Renal Insufficiency Cohort (CRIC) Study [11, 21], the Lund Cystatin C Standardization (LCS) Cohort [17], the Incidence, Development, and Prognosis of Diabetic Kidney Disease (INDEED) Study [34], the National Health and Nutrition Examination Survey (NHANES) [34], the UK Biobank [34, 35], the Korean Cohort Study for Outcome in Patients With Chronic Kidney Disease (KNOW-CKD) [39], the Systolic Blood Pressure Intervention Trial (SPRINT) Study [42], the Northern Sweden Health and Disease Study (NSHDS) [43] and the HARVEST-Malmo Study [45]. Four studies focused on the general population, including people from the Atherosclerosis Risk in Communities (ARIC) Study [22], the Stockholm Creatinine Measurements (SCREAM) project [33], the UK Biobank [36] and the Malmo Osteoporosis Prospective Risk Assessment (OPRA) cohort [41]. Study populations self-collected by researchers were all patients, including those with fractures [38], individuals who received cardiac surgery [37, 40, 46] and patients with kidney disease [44].

In selected cohort studies, the median follow-up time differed among the studies based on the outcome (e.g. the follow-up time for CVD incidence is generally shorter than that for mortality). Two studies did not report the median follow-up time [38, 42], while two others provided only the minimum follow-up time [22, 41]. For the remaining studies, the median follow-up time ranged from 1.8 to 15.17 years, with an overall median of 5.2 years. Except for one study with an attrition rate of 20%, all the other studies have a low attrition rate [39].

### Measurement of eGFR and eGFR discordances

Except for 3 studies [38, 40, 43] that solely applied the eGFR equation based on Caucasian, Asian, Paediatric, and Adult cohorts (CAPA) [47] and the Lund-Malmö revised creatinine-based eGFR equation (LMrev) [48] to calculate eGFR discordance, the remaining 15 studies applied the Chronic Kidney Disease Epidemiology Collaboration (CKD-EPI) equations; 1 study applied both race-independent and race-free CKD-EPI equations [36].

Of the 15 studies that applied CKD-EPI equations, 8 used the latest 2021 race-free eGFRcr equation [49]. Among the remaining seven articles, four studies were published before or around the time the race-free equation was released, which explained why they did not use the latest equation [17, 39, 42, 45]. However, for the other three studies, there was no clear explanation [37, 41, 44]. Additionally, four studies used both the CAPA-LMrev and CKD-EPI equations [17, 37, 41, 44], and one of these studies [17] also applied the full age spectrum (FAS) equation [50]. Studies that used the CAPA-LMrev equation were all conducted on Nordic people [17, 37, 38, 40, 41, 43] except one which was on a Chinese population [44].

The definition of eGFR discordance in the selected studies was based on absolute differences and/or relative differences. Studies using absolute differences assessed the effects of eGFR discordance in both directions, i.e. when eGFRcys was either lower or higher than eGFRcr. In contrast, studies using relative values to measure eGFR discordance predominantly assessed the effect when eGFRcys was less than eGFRcr, with two studies examined the effect in both directions [22, 33]. All the selected studies, except for one that used eGFRcr as the minuend [39], used eGFRcr as the subtrahend or denominator.

The cutoff values for eGFR discordance were largely consistent across studies. For the absolute difference, most studies considered eGFRcys-eGFRcr <–15 mL/min/1.73 m^2^ and >15 mL/min/1.73 m^2^ as indicative of eGFR discordance, with eGFRcys-eGFRcr between –15 and 15 mL/min/1.73 m^2^ serving as the reference group. The rationale for using 15 mL/min/1.73 m^2^ as the cutoff value is that it approximates 1 SD in most populations and signifies a clinically meaningful difference that indicates CKD stages [21]. One study evaluated eGFR discordance based on tertiles of eGFRcys-eGFRcr [39], while another study used the quartile of the same difference [33]. For the relative difference, most studies defined eGFR discordance as eGFRcys being less than or equal to 60% or 70% of eGFRcr. One study explored health effects when eGFRcys was 30% higher than eGFRcr [22]. Another study used the quartiles of the percentage difference between eGFRcys and eGFRcr, defined as (eGFRcys – eGFRcr)/eGFRcr [33].

### Measurement of outcomes

Among the included studies, 11 studies have mortality as the research outcome [11, 17, 22, 33, 34, 37, 38, 41, 42, 45, 46]. Mortality included both long-term and short-term mortality (e.g. 90-day mortality). Most studies reported all-cause mortality, while one study specifically examined cause-specific mortality [17].

Five studies discussed the association between eGFR discordance and long-term kidney outcomes, including kidney failure, ESKD, severe eGFR decline [serum creatinine (SCr) doubling or ≥50% eGFRcr decline compared with the baseline] and diabetic kidney disease (DKD) [11, 22, 33, 35, 44]. Additionally, three studies investigated acute kidney injury (AKI) [22, 33, 46]. Research outcomes were confirmed through patient records or database linkage.

Of the 10 studies that used CVD incidence as an outcome [21, 22, 33, 34, 36, 39, 40, 42, 43, 45], 1 focused solely on heart failure (HF) [21], 1 reported results for HF and atherosclerotic CVD separately [33], 1 studied aortic stenosis [40] and 1 focused on myocardial infarction [43], while the remaining studies combined multiple CVDs, including myocardial infarction, atrial fibrillation, stroke and HF.

### Potential bias and quality assessment

The overall quality of the selected studies was good. The ROBINS-E overall scale was ‘Low’ or ‘Some concerns’ in 13 out of 18 studies. Four studies were categorized as high risk of bias because their insufficient adjustments for confounding effect [17, 37, 40, 43]. One study was graded as of very high risk of bias because its authors used CAPA and LMrev equations in defining study exposure but then used CKD-EPI equations in follow-up assessments in primary analysis (Table 3) [44].

**Table 3:** ROBINS-E assessment form.

Author, year [Ref.]	Risk of bias due to confounding	Risk of bias arising from measurement of the exposure	Risk of bias in selection of participants into the study (or into the analysis)	Risk of bias due to post-exposure interventions	Risk of bias due to missing data	Risk of bias arising from measurement of the outcomes	Risk of bias in selection of the reported results	Overall
Åkesson *et al*. 2020 [17]	High	Low	Low	Low	Low	Low	Low	High
Carrero *et al*. 2023 [33]	Some concerns	Low	Low	Low	Low	Low	Low	Low
Chen *et al*. 2022a [11]	Low	Low	Low	Low	Low	Low	Low	Low
Chen *et al*. 2022b [21]	Low	Low	Low	Low	Low	Low	Low	Low
Farrington *et al*. 2023 [22]	Low	Some concerns	Low	Low	Some concerns	Low	Low	Some concerns
He *et al*. 2024a [34]	Low	Low	Low	Low	Some concerns	Low	Low	Some concerns
He *et al*. 2024b [35]	Low	Low	Low	Low	Low	Low	Low	Low
Heo *et al*. 2024 [36]	Low	Low	Low	Low	Low	Some concerns	Low	Some concerns
Herou *et al*. 2022 [37]	High	Low	Low	Low	Low	Low	Low	High
Jonsson *et al*. 2021 [38]	Some concerns	Low	Low	Low	Low	Low	Low	Some concerns
Kim *et al*. 2021 [39]	Low	Low	Low	Low	Some concerns	Low	Low	Some concerns
Ljungberg *et al*. 2019 [40]	High	Low	Low	Low	Low	Low	Low	High
Malmgren *et al*. 2022 [41]	Low	Low	Low	Low	Low	Low	Low	Low
Potok *et al*. 2020 [42]	Low	Low	Low	Low	Low	Low	Low	Low
Söderström *et al*. 2021 [43]	High	Low	Low	Low	Low	Low	Low	High
Wu *et al*. 2022 [44]	Low	Very high	Low	Low	Low	Very high	Low	Very high
Xhakollari *et al*. 2021 [45]	Some concerns	Low	Low	Low	Low	Low	Low	Some concerns
Zhang *et al*. 2023 [46]	Some concerns	Low	Low	Low	Low	Low	Low	Some concerns

Aligned with Table 3, the main risk of bias was due to confounding, followed by missing data. Measurement of exposure and outcome were generally accurate (Supplementary data, Figs S1 and S2).

### Systematic review on the association of eGFR discordances with mortality

Across 11 studies, mortality was elevated when eGFRcys was less than eGFRcr [11, 17, 22, 33, 34, 37, 38, 41, 42, 45, 46]. Compared with people with eGFRcys-eGFRcr within ±15 mL/min/1.73 m^2^, those with lower eGFRcys-eGFRcr showed an elevated risk of mortality. For each 1 SD increase in eGFRcys-eGFRcr, there was a significant decrease in the risk of mortality. People with an eGFRcys/eGFRcr ≤0.6 or 0.7 have elevated mortality, compared with those with a higher ratio. Studies using CKD-EPI, CAPA-LMrev and FAS equations have similar results [17, 37, 38, 41].

Eight studies conducted stratified analyses [11, 17, 22, 33, 34, 37, 42, 45], and only a few studies tested for interaction effects. Two studies found that the association between eGFR discordance and mortality was significant for both sexes, although no interaction with sex was observed [11, 34]. Another study also showed no interactions of sex, but subgroup results were not presented [42]. A fourth study observed the interaction effect of sex on the association of eGFR discordance with all-cause mortality, but not with CVD mortality [33].

Three studies tested the interaction effect of age, using 60 or 65 years as the threshold, with inconsistent results. In Chen *et al*.’s study on the CRIC cohort [11], and He *et al*.’s study on the INDEED cohort [34], no interaction was found. However, an interaction of age was observed in the NHANES and UK Biobank populations in the same study by He *et al*. [34]. The study by Carrero *et al*. showed consistent interaction effect of age that people aged 65 years or above have attenuated effect size [33].

Five studies performed eGFR-stratified analyses based on eGFR calculated using CKD-EPI equations [11, 22, 33, 34, 37]. Various eGFR cutoff values were applied, including 45 and 90 mL/min/1.73 m^2^ [11, 34], and by CKD stages [22, 33, 37]. The interaction effect of baseline eGFR was inconsistent. In a subset of the UK Biobank population, a consistent significant interaction was found; people with baseline eGFR <90 mL/min/1.73 m^2^ have a smaller effect size [34]. Similar findings were also presented in another study where patients with a higher CKD stage. One study showed that Black people have lesser risk (*P* for interaction = .042) [11], but another study did not [42].

### Meta-analysis on the association of eGFR discordances with all-cause mortality

Three studies were not included in the meta-analysis because their reference groups were different from that of other studies thus incomparable [17, 33, 41]. Among the remaining studies, only one study focused on community dwellers [22]. Thus, a meta-analysis for the general population could not be conducted. Subject to data consistency, only studies using the CKD-EPI eGFR equation were used in the meta-analysis. Studies using different versions of CKD-EPI equations were jointly analysed for simplicity.

One study used an eGFRcys/eGFRcr <0.6 as the criterion for eGFR discordance [34], while the other three studies used a ratio of ≤0.6 [37, 45, 46]. To simplify the analysis, we combined these studies. We regarded the slight difference in the definition of eGFR discordance had a minimal impact on the study results and was therefore acceptable.

**Figure 2:**
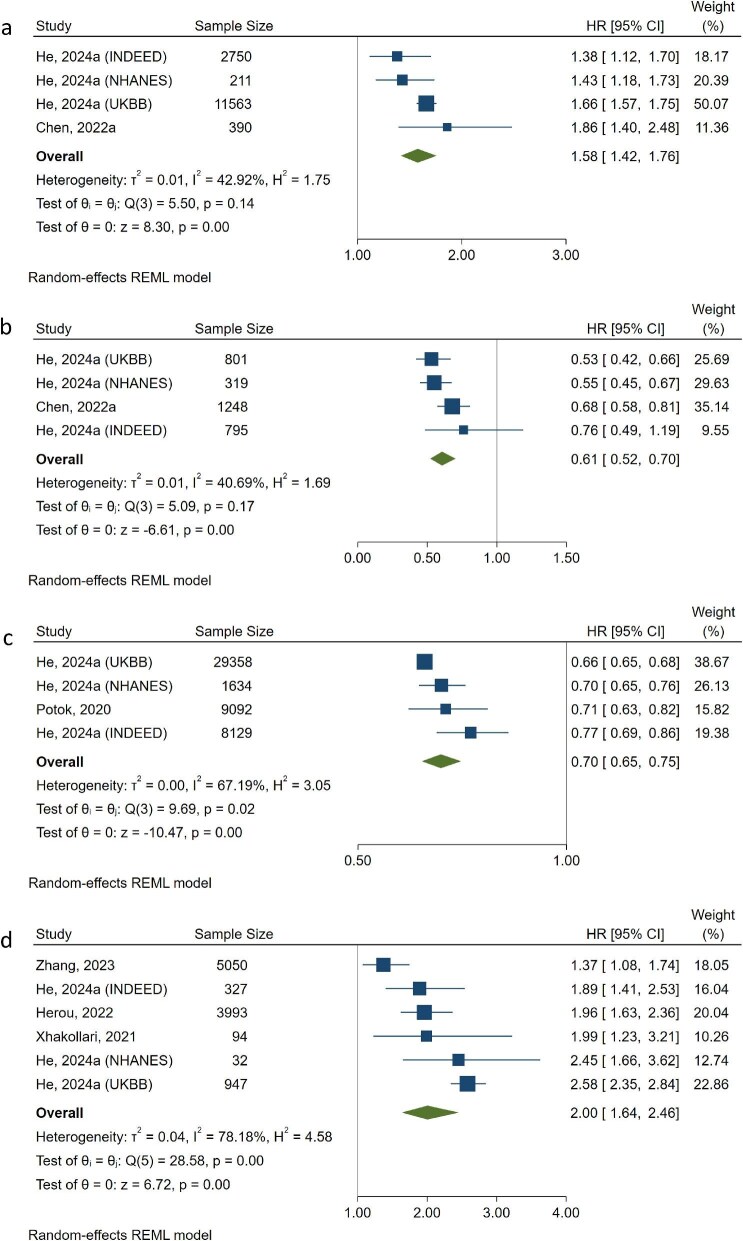
Forest plot of meta-analysis on the association between eGFR discordance and mortality. (**a**) eGFR discordance was defined as eGFRcys-eGFRcr <–15 mL/min/1.73 m^2^. (**b**) eGFR discordance was defined as eGFRcys-eGFRcr ≥15 mL/min/1.73 m^2^. (**c**) Per 1 SD increase of eGFRcys-eGFRcr. (**d**) eGFR discordance was defined as eGFRcys/eGFRcr ≤0.6.

With a total population of 19 414 people from two studies [11, 34], the pooled result showed a significant positive association of eGFR discordance with mortality when eGFRcys-eGFRcr below –15 mL/min/1.73 m^2^, compared with people with eGFRcys-eGFRcr between ±15 mL/min/1.73 m^2^ (HR = 1.58, 95% CI 1.42, 1.76). Heterogeneity among studies was moderate (I^2^ = 42.92%, 95% CI 22.93%, 62.91%). Leave-one-out analyses showed positive associations, funnel plot showed possible bias that studies with smaller sizes tend to report larger effect sizes (Fig. 2a, Supplementary data, Figs S3a and S4a).

The pooled result of the association of eGFR discordance with mortality when eGFRcys-eGFRcr was ≥15 mL/min/1.73 m^2^ in a total population of 3163 people showed significance [11, 34]. Those people have a 39% lower risk of mortality (HR = 0.61, 95% CI 0.52, 0.70) as compared with those with eGFRcys-eGFRcr between ±15 mL/min/1.73 m^2^, with moderate heterogeneity (I^2^ = 40.69%, 95% CI 19.36%, 62.02%). Leave-one-out analyses showed consistent effects and potential bias was found (Fig. 2b, Supplementary data, Figs S3b and S4b).

Treating eGFRcys-eGFRcr as a continuous variable, the pooled result in a total population of 48 213 people showed per 1 SD elevation of eGFRcys-eGFRcr was associated with a 30% lower mortality risk (HR = 0.70, 95% CI 0.65, 0.75) [34, 42]. Heterogeneity was substantial (I^2^ = 67.19%, 95% CI 56.24%, 78.14%). Leave-one-out analyses showed a consistent effect. Serious bias was found that small sample-size studies were more likely to underestimate the effect (Fig. 2c, Supplementary data, Figs S3c and S4c).

Using eGFRcys/eGFRcr to denote eGFR discordance, in a total population of 10 443 people with eGFRcys/eGFRcr ≤0.6, the pooled result showed a doubled risk of mortality (HR = 2.00, 95% CI 1.64, 2.46) [34, 37, 45, 46], albeit with substantial heterogeneity (I^2^ = 78.18%, 95% CI 73.90%, 82.46%). Leave-one-out analyses showed a consistent effect, small sample-size studies were likely to overestimate the effect (Fig. 2d, Supplementary data, Figs S3d and S4d).

Subject to data availability, only stratified meta-analysis on median C-reactive protein (CRP) levels and obesity [body mass index (BMI) ≥30 kg/m^2^] were performed. In people with eGFRcys-eGFRcr <–15 mL/min/1.73 m^2^, those with CRP ≥2 mg/L have a stronger association of discordance with mortality compared with those with lower CRP levels (HR = 1.67, 1.41, respectively), the between-group difference was significant (*P* = .03) (Supplementary data, Fig. S5a and b). People with obesity have a significantly higher pooled effect of eGFRcys/eGFRcr ≤0.6 on mortality compared with non-obese counterparts (*P* < .001, HR = 2.57, 1.66, respectively) (Supplementary data, Fig. S5c).

### Studies on the association of eGFR discordances with kidney outcomes

The association of eGFR discordances with kidney outcomes was measured by several models, including Cox regression [22, 33, 35, 44], logistic regression [46] and Fine–Gray proportional subhazards model with mortality as a competing risk [11, 35]. One study also presented findings using time-updated covariates [11].

In general, eGFR discordance was significantly associated with the incidence of kidney outcomes. Compared with people with similar eGFRcys and eGFRcr, people with eGFRcys-eGFRcr <–15 mL/min/1.73 m^2^ have 63% higher risk of DKD incidence (HR = 1.63, 95% CI 1.50, 1.76), and those with eGFRcys-eGFRcr ≥15 mL/min/1.73 m^2^ have 61% lower risk of DKD incidence (HR = 0.39, 95% CI 0.31, 0.50), with similar findings in Fine–Gray models [35].

The association between eGFR discordance and ESKD incidence was only significant when eGFRcys-eGFRcr was modelled as a time-updated covariate, people with eGFRcys-eGFRcr <–15 mL/min/1.73 m^2^ had 83% elevated risk of ESKD [subhazard ratio (sHR) = 1.83, 95% CI 1.10, 3.04], while those with eGFRcys-eGFRcr ≥15 mL/min/1.73 m^2^ have the risk dropped by 50% (sHR = 0.50, 95% CI 0.35, 0.71) [11]. One study showed that people in the lowest quartile of eGFRcys-eGFRcr have 210% elevated risk of AKI (HR = 3.10, 95% CI 2.85, 3.36) and 36% elevated risk of kidney failure with replacement therapy (HR = 1.36, 95% CI 1.17, 1.58), compared with those with similar eGFRcys and eGFRcr [33].

When eGFR discordance was defined as eGFRcys/eGFRcr ≤0.6 or <0.7, its association with DKD, ESKD and AKI was significantly positive [22, 35, 46]. Similar findings were observed when the quartile of the percentage difference between eGFRcys and eGFRcr was used [33]. No association with severe eGFR decline was found (HR = 1.87, 95% CI 0.86, 4.06) in females with immunoglobulin A (IgA) nephropathy or membranous nephropathy during a median follow-up of 2.3 years [44].

Four studies conducted stratified analyses and all have interaction effects tested [11, 33, 35, 46]. In three studies, no interaction of age, sex, Black ethnicity, diabetes and hypertension was found [11, 35, 46]. Yet, in one large-scale study (*N* = 158 601), there was a consistent interaction effect of age, baseline eGFR category, and hypertension on the association of eGFR discordance with kidney failure / AKI [33]. Compared with younger individuals, those over 65 years of age have a lower risk of renal outcomes. Similarly, individuals with lower baseline eGFR (as calculated using the CKD-EPI equation), or those with hypertension, have a reduced renal risk compared with those with higher baseline eGFR or without hypertension [33]. There was a significant interaction (*P* for interaction <.05) of CVD history on the association between eGFR discordance and DKD incidence; people with historical CVD have a lower risk, but no subgroup values were reported [35].

Limited by outcome data, no meta-analysis on the association of eGFR discordance with kidney outcomes was performed.

### Studies on the association of eGFR discordance with cardiovascular outcomes

The association of eGFR discordance with cardiovascular outcomes has been explored in classical Cox regression [22, 33, 34, 42, 45], cause-specific Cox regression [39], logistic regression [40, 43] and Fine–Gray proportional subhazards model with mortality as a competing risk [21, 34, 36].

Compared with individuals with similar eGFRcys and eGFRcr, those with lower eGFRcys-eGFRcr have an elevated risk of CVD incident in general [21, 33, 34, 36], despite a few discrepancies among cohorts [34]. Those with higher eGFRcys-eGFRcr have a lower risk of CVD incidents, although cohort discrepancies existed [21, 33, 34, 36]. Per 1 SD greater in eGFRcys-eGFRcr has a consistent negative association with CVD incidence [34, 42]. A study showed that people in the highest tertile of eGFRcr-eGFRcys had an elevated incidence of major adverse cardiovascular events (MACE), indirectly confirming that a higher eGFRcys-eGFRcr was associated with a lower incidence [39].

People with eGFRcys/eGFRcr ≤0.6 or <0.7 have an elevated risk of HF and 30-day rehospitalization due to cardiac causes, compared with those with a higher eGFRcys/eGFRcr ratio [22, 45]. Two studies showed people in the highest quartile of eGFRcys/eGFRcr (i.e. higher eGFRcys/eGFRcr) have a reduced risk of surgery for aortic stenosis and a lower risk of myocardial infarction in women, compared with those in the lowest quartile of eGFRcys/eGFRcr [40, 43].

Nine studies conducted stratified analyses [21, 33, 34, 36, 39, 40, 42, 43, 45], among which six studies have interaction test results available [21, 33, 34, 36, 39, 42]. The interaction of age, baseline eGFR (by CKD-EPI equation) and sex was not found in most analyses, except in a few studies [33, 34, 39, 42]. Specifically, an interaction of age was observed when the cutoff value was set at 65 or 75 years, but not at 60 years. In individuals aged 65 or 75 years and above, the effect of eGFR discordance was attenuated [33, 34, 42]. Females (*P* for interaction = .016) [39] and those with baseline eGFR above 90 mL/min/1.73 m^2^ (*P* for interaction = .035) [34] have a higher risk of MACE. No consistent interactions of albuminuria, BMI, hypertension, obesity and race were found [21, 33, 34, 36, 39, 42].

### Meta-analysis on the association of eGFR discordance with cardiovascular outcomes

Although three studies were conducted on the general population, a meta-analysis could not be performed because their eGFR discordance was measured differently [22, 33, 36]. Another study was excluded from the meta-analysis because it reported a cause-specific HR that could not be compared with other studies [39]. Two cross-sectional studies were also excluded because their outcomes were not comparable with each other [40, 43]. Studies that reported both Fine–Gray sHRs and Cox HRs had these ratios synthesized separately, with the pooled results of the Fine–Gray sHRs presented in the main text.

**Figure 3:**
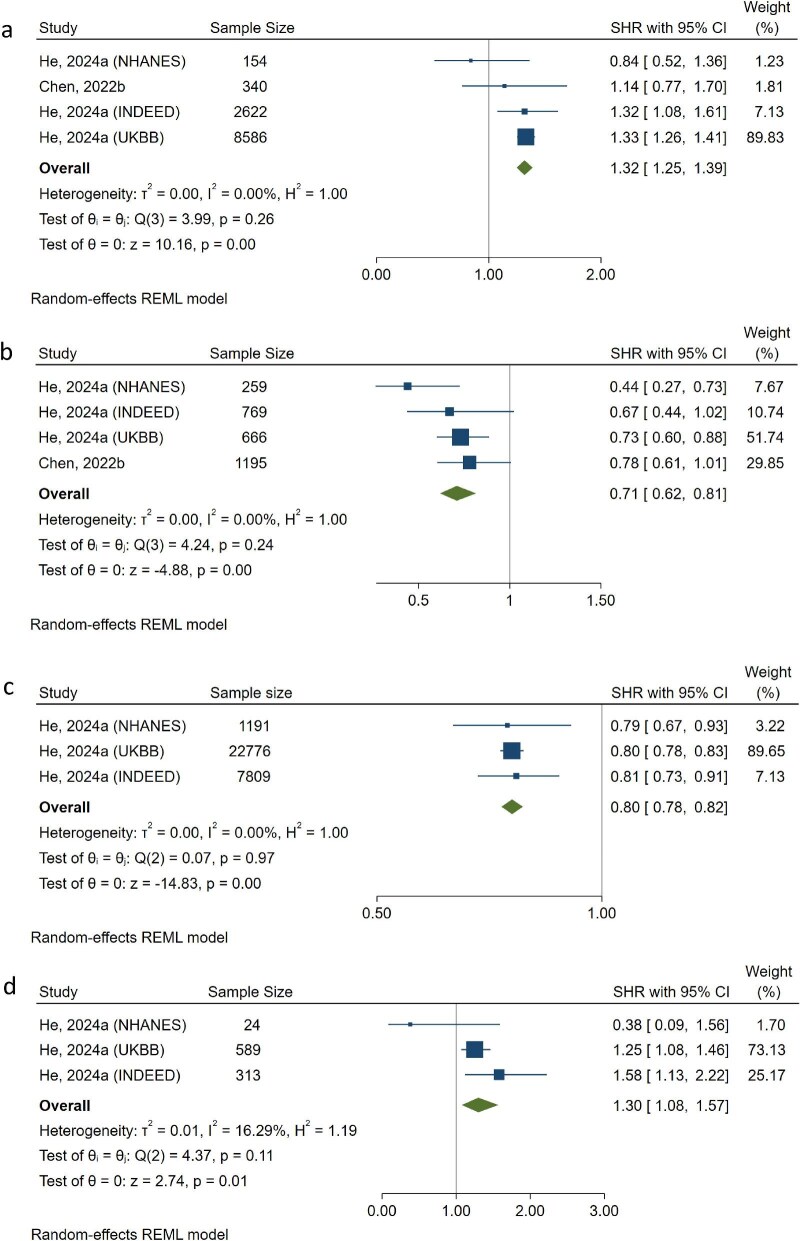
Forest plot of meta-analysis on the association between eGFR discordance and cardiovascular incidence, Fine–Gray competing risk model. (**a**) eGFR discordance was defined as eGFRcys-eGFRcr <–15 mL/min/1.73 m^2^. (**b**) eGFR discordance was defined as eGFRcys-eGFRcr ≥15 mL/min/1.73 m^2^. (**c**) Per 1 SD increase of eGFRcys-eGFRcr. (**d**) eGFR discordance was defined as eGFRcys/eGFRcr ≤0.6.

With a total population of 11 702 people from two studies [21, 34], the pooled result showed a significantly elevated risk of CVD incidences in people with eGFRcys-eGFRcr <–15 mL/min/1.73 m^2^, compared with those with similar eGFRcys and eGFRcr (HR = 1.32, 95% CI 1.25, 1.39). Heterogeneity among studies was small (I^2^ = 0.00%, 95% CI 0.00%, 23.79%). Leave-one-out analyses showed the association would be nullified if either of the two largest study populations was omitted. Studies with small sample sizes tended to underestimate the association (Fig. 3a, Supplementary data, Figs S6a and S7a).

The pooled 2889 people with eGFRcys-eGFRcr ≥15 mL/min/1.73 m^2^ have a pooled result of 29% lower risk of CVD incidence (HR = 0.71, 95% CI 0.62, 0.81) compared with those with similar eGFRcys and eGFRcr [21, 34]. Heterogeneity among studies was small (I^2^ = 0.00%, 95% CI 0.00%, 23.66%). Leave-one-out analyses showed consistent associations. Studies with small sample sizes tended to underestimate the association (Fig. 3b, Supplementary data, Figs S6b and S7b). In addition, the pooled results of three populations from a single study showed that per 1 SD increase in eGFRcys-eGFRcr was associated with a 20% lower risk of CVD incidence (HR = 0.80, 95% CI 0.78, 0.82) [34]. Heterogeneity among studies was small (I^2^ = 0.00%, 95% CI 0.00%, 39.91%). Results were less likely to be biased and were consistent in leave-one-out analyses (Fig. 3c, Supplementary data, Figs S6c and S7c).

In the pooled population of 926 people with eGFRcys/eGFRcr <0.6, they have a 30% more risk (HR = 1.30, 95% CI 1.08, 1.57) of CVD incidence compared with those with higher eGFRcys/eGFRcr ratio [34]. Heterogeneity was low (I^2^ = 16.29%, 95% CI 0.00%, 32.82%). Yet, in leave-one-out analyses, the results were insignificant if either of the two largest study populations was excluded. Small-size study populations were likely to underestimate the association (Fig. 3d, Supplementary data, Figs S6d and S7d). Studies reported Cox hazard ratio has similar pooled results (Supplementary data, Figs S8a–d, S9a–d and S10a–d). No stratified meta-analysis was conducted due to data availability.

## DISCUSSION

Overall, this meta-analysis affirms that eGFR discordance characterized by eGFRcys being lower than eGFRcr is common and is positively and consistently associated with adverse health outcomes, including mortality, ESKD and HF incidence. Conversely, eGFR discordance characterized by eGFRcys being higher than eGFRcr is significantly associated with lower mortality and CVDs. The interaction effects of sex, age, race and comorbidities remained inconclusive, and most studies have reported insignificant interactions. The association between eGFR discordance and mortality was enhanced by higher CRP levels (cutoff: 2 mg/L, *P* for interaction <.001), which is a factor not previously addressed in selected studies.

Our observations suggest that people with a large discordance between eGFRcys and eGFRcr have a different pattern of risks on mortality, CVD events and renal events, compared with people with similar eGFRcys and eGFRcr. Current research suggests that eGFR discordance may arise from a reduced pore diameter in the glomerular basement membrane, making it more difficult for cystatin C to be filtered while smaller molecules like creatinine pass through easily [12, 15]. This results in the accumulation of circulating levels of cystatin C [13]. In patients with SPS, proteins with molecular weights similar to cystatin C, despite having almost completely different functions, have their filtration impeded, resulting in elevated levels [51]. This indicates that eGFR discordance may at least partly be caused by physical, rather than chemical, factors.

Non-GFR factors are assumed to play a key role in explaining the association between eGFR discordance and health outcomes. Declining health may lead to reduced physical activity, leading to muscle loss and decreased creatinine production, which may prevent changes in eGFRcr from falling alongside declining kidney function. In contrast, the impact of declining health on cystatin C is independent of muscle mass, though may be related to accrual of metabolic disease, resulting in a more linear decrease in eGFRcys. As these factors are more likely to occur with increasing age, people with the lowest eGFRcys/eGFRcr ratio tend to be older [33, 34, 42] and have a higher prevalence of sarcopenia [34]. Sarcopenia is associated with multiple adverse health outcomes, including frailty and disability, and often with other age-related conditions like osteoporosis, CVD and metabolic disorders—all of which contribute to increased morbidity and mortality in older adults [52]. It is characterized by the reduction in muscle mass which leads to lower creatinine production, potentially resulting in eGFRcr overestimating actual kidney function. Sharma *et al*. showed that in obese people (defined as total body fat% above the sex-specific 60th percentile, measured using dual-energy X-ray absorptiometry) with sarcopenia, 97.7% were misclassified as not obese by BMI [53]. Therefore, non-GFR factors may be a key contributor in the association between eGFR discordance and health outcomes, especially in older adults.

Additionally, studies have found that people with an eGFRcys/eGFRcr ratio <0.6 have higher levels of proteins related to atherosclerosis and cell proliferation, such as IL-6, CXCL10 and FGF23 [51]. These proteins are known risk factors for mortality, kidney disease and CVD [54–56].

Notably, in a study presented iohexol-measured measured GFR (mGFR), the mGFR category (<30, 30–59 and 60–89 mL/min/1.73 m^2^, respectively; reference group = 90 mL/min/1.73 m^2^) was not the risk factor for all-cause mortality [17]. Instead, a low eGFRcys/eGFRcr ratio defined by the CAPA-LMrev equations was a risk factor (reference group = ratio ≥1.0) in the same study [17]. This further suggests that eGFR discordance may indicate the influence of non-GFR factors.

A notable limitation in several selected studies is the inadequate adjustment for confounding factors. Several known factors of creatinine levels and renal health, such as muscle mass, sarcopenia and albuminuria, were not included as explanatory variables [17, 37, 40, 43]. While insufficient adjustment for confounding factors is a recognized disadvantage of using existing study populations, inadequate adjustment may have inflated eGFR discordances, particularly in studies with small sample sizes [40, 43].

Another issue that is worth attention comes from the large difference in the follow-up duration of cohort studies. In the meta-analysis of all-cause mortality, the median of follow-up duration ranged from 3.83 to 15.17 years [11, 34]. In the meta-analysis of CVD events, the median of follow-up duration ranged from 3.5 to 15.92 years [21, 34]. This variability makes it difficult to distinguish the short- and long-term effects of eGFR discordance, as shorter durations may miss late-onset events and chronic exposure effects.

The standard for eGFR discordance has not been uniformly defined, and the current standard is based on consensus rather than evidence-based approaches. The prevalence of eGFR discordance can vary significantly depending on the equations used (e.g. 11.5% with the CKD-EPI equation and 2.3% with the CAPA-LMrev equation) [17], although the association between eGFR discordance and health outcomes remains consistent [37]. It is worth investigating whether to define different eGFR discordance criteria for different equations or to develop an equation-independent criterion.

Several studies have used both eGFRcys-eGFRcr and eGFRcys/eGFRcr to assess eGFR discordance. A recent study demonstrated that these two approaches have nearly identical predictive accuracy for mortality, as reflected by similar Harrell's C-index values [57]. However, they classified different subsets of individuals as having discordant eGFR values, which may reflect variations in the underlying non-GFR factors influencing the results. For example, 6.2% (172 out of 2781) of the study population had an eGFRcys-eGFRcr between 0 and –15 mL/min/1.73 m^2^ and an eGFRcys/eGFRcr <0.7, highlighting differences between the two approaches. Whether these differences impact outcomes beyond mortality or reflect specific pathophysiological processes remains uncertain, as insufficient data exist to determine whether eGFRcys or eGFRcr better reflects mGFR in this subgroup.

Therefore, future research on eGFR discordance may seek to: (i) establish a consensus on the definition of eGFR discordance; (ii) discuss the possible role of eGFR discordance as a mediator on the pathway between other known risk factors; (iii) explore the extent of eGFR discordance that can explain the residual risks after adjusting for known risk factors; and (iv) explore the association between eGFR discordance and health outcomes in the general population.

The limitations of the study include the following. (i) Some studies did not adequately adjust for confounding factors, which may lead to biased results. (ii) Due to data availability, our meta-analysis focused only on eGFR discordance measured by the CKD-EPI equation. This limits the generalizability of findings. (iii) A meta-analysis on the association between eGFR discordance and kidney outcomes was not performed, affecting the comprehensive evaluation of kidney outcomes. (iv) Due to data availability, we did not conduct subgroup meta-analyses on important variables such as sex and age, restricting our understanding of the effect of eGFR discordance in different populations. (v) Follow-up lengths of selected studies vary significantly, which may affect the results. This variation could lead to heterogeneity, impacting the comparability and interpretation of findings. (vi) The study included primarily populations with specific diseases, primarily from Europe and the USA, which may not be applicable to the general population or other populations. (vii) Grey literature was excluded from this review. Although this exclusion might only bring very limited impact to the conclusions, if any, readers should be aware of potential alternation to our findings.

In conclusion, our study thoroughly explored the association between eGFR discordance and adverse health outcomes, providing the first systematic insights to our best knowledge. Despite certain limitations, our research, based on high-quality studies and comparative analyses of various eGFR discordance measurement methods, may lay the groundwork for further understanding the clinical significance and practical value of eGFR discordance.

## Supplementary Material

sfaf003_Supplemental_File

## Data Availability

The data underlying this article will be shared on reasonable request to the corresponding author.

## References

[1] Groothof D, Post A, Polinder-Bos HA et al. Muscle mass and estimates of renal function: a longitudinal cohort study. J Cachexia Sarcopenia Muscle 2022;13:2031–43. 10.1002/jcsm.12969 35596604 PMC9398222

[2] Nankivell BJ, Nankivell LFJ, Elder GJ et al. How unmeasured muscle mass affects estimated GFR and diagnostic inaccuracy. EClinicalMedicine 2020;29-30:100662. 10.1016/j.eclinm.2020.100662 33437955 PMC7788434

[3] Baxmann AC, Ahmed MS, Marques NC et al. Influence of muscle mass and physical activity on serum and urinary creatinine and serum cystatin C. Clin J Am Soc Nephrol 2008;3:348–54. 10.2215/CJN.02870707 18235143 PMC2390952

[4] Kleeman SO, Thakir TM, Demestichas B et al. Cystatin C is glucocorticoid responsive, directs recruitment of Trem^2^+ macrophages, and predicts failure of cancer immunotherapy. Cell Genomics 2023;3:100347. 10.1016/j.xgen.2023.100347 37601967 PMC10435381

[5] Rule AD, Bailey KR, Lieske JC et al. Estimating the glomerular filtration rate from serum creatinine is better than from cystatin C for evaluating risk factors associated with chronic kidney disease. Kidney Int 2013;83:1169–76. 10.1038/ki.2013.7 23423253 PMC3661736

[6] Inker LA, Levey AS, Coresh J. Estimated glomerular filtration rate from a panel of filtration markers—hope for increased accuracy beyond measured glomerular filtration rate? Adv Chronic Kidney Dis 2018;25:67–75. 10.1053/j.ackd.2017.10.004 29499889

[7] Shlipak MG, Matsushita K, Ärnlöv J et al. Cystatin C versus creatinine in determining risk based on kidney function. N Engl J Med 2013;369:932–43. 10.1056/NEJMoa1214234 24004120 PMC3993094

[8] Helmersson-Karlqvist J, Lipcsey M, Ärnlöv J et al. Addition of cystatin C predicts cardiovascular death better than creatinine in intensive care. Heart 2022;108:279–84. 10.1136/heartjnl-2020-318860 33795382 PMC8819658

[9] Lees JS, Rutherford E, Stevens KI et al. Assessment of cystatin C level for risk stratification in adults with chronic kidney disease. JAMA Netw Open 2022;5:e2238300. 10.1001/jamanetworkopen.2022.38300 36282503 PMC9597396

[10] Jernberg T, Lindahl B, James S et al. Cystatin C: a novel predictor of outcome in suspected or confirmed non-ST-elevation acute coronary syndrome. Circulation 2004;110:2342–8. 10.1161/01.CIR.0000145166.44942.E0 15477399

[11] Chen DC, Shlipak MG, Scherzer R et al. Association of intraindividual difference in estimated glomerular filtration rate by creatinine vs Cystatin C and end-stage kidney disease and mortality. JAMA Netw Open 2022;5:e2148940. 10.1001/jamanetworkopen.2021.48940 35175342 PMC8855239

[12] Quiroga B, Ortiz A, Díez J. Selective glomerular hypofiltration syndrome. Nephrol Dial Transplant 2023;39:10–17. 10.1093/ndt/gfad145 37407284

[13] Grubb A . Shrunken pore syndrome—a common kidney disorder with high mortality. Diagnosis, prevalence, pathophysiology and treatment options. Clin Biochem 2020;83:12–20. 10.1016/j.clinbiochem.2020.06.002 32544475

[14] Grubb A, Lindström V, Jonsson M et al. Reduction in glomerular pore size is not restricted to pregnant women. Evidence for a new syndrome: ‘Shrunken pore syndrome’. Scand J Clin Lab Invest 2015;75:333–40. 10.3109/00365513.2015.1025427 25919022 PMC4487590

[15] Malmgren L, Öberg C, den Bakker E et al. The complexity of kidney disease and diagnosing it—cystatin C, selective glomerular hypofiltration syndromes and proteome regulation. J Intern Med 2023;293:293–308. 10.1111/joim.13589 36385445 PMC10107454

[16] Liu C, Levey AS, Ballew SH. Serum creatinine and serum cystatin C as an index of muscle mass in adults. Curr Opin Nephrol Hypertens 2024;33:557–65. 10.1097/MNH.0000000000001022 39155834

[17] Åkesson A, Lindström V, Nyman U et al. Shrunken pore syndrome and mortality: a cohort study of patients with measured GFR and known comorbidities. Scand J Clin Lab Invest 2020;80:412–22. 10.1080/00365513.2020.1759139 32459111

[18] Dardashti A, Nozohoor S, Grubb A et al. Shrunken Pore Syndrome is associated with a sharp rise in mortality in patients undergoing elective coronary artery bypass grafting. Scand J Clin Lab Invest 2016;76:74–81. 10.3109/00365513.2015.1099724 26647957 PMC4720044

[19] Purde M-T, Nock S, Risch L et al. Ratio of cystatin C and creatinine-based estimates of the glomerular filtration rate predicts mortality in healthy seniors independent of kidney function. Scand J Clin Lab Invest 2016;76:341–3. 10.3109/00365513.2016.1149882 26981764

[20] Purde MT, Nock S, Risch L et al. The cystatin C/creatinine ratio, a marker of glomerular filtration quality: associated factors, reference intervals, and prediction of morbidity and mortality in healthy seniors. Transl Res 2016;169:80–90. e1–2. 10.1016/j.trsl.2015.11.001 26637934

[21] Chen DC, Shlipak MG, Scherzer R et al. Association of intra-individual differences in estimated GFR by creatinine versus cystatin C with incident heart failure. Am J Kidney Dis 2022;80:762–72.e1. 10.1053/j.ajkd.2022.05.011 35817274 PMC9691565

[22] Farrington DK, Surapaneni A, Matsushita K et al. Discrepancies between Cystatin C-based and creatinine-based eGFR. Clin J Am Soc Nephrol 2023;18:1143–52. 10.2215/CJN.0000000000000217 37339177 PMC10564370

[23] Page MJ, McKenzie JE, Bossuyt PM et al. The PRISMA 2020 statement: an updated guideline for reporting systematic reviews. BMJ 2021;372:n71. 10.1136/bmj.n71 33782057 PMC8005924

[24] Stroup DF, Berlin JA, Morton SC et al. Meta-analysis of observational studies in epidemiology: a proposal for reporting. Meta-analysis Of Observational Studies in Epidemiology (MOOSE) group. JAMA 2000;283:2008–12. 10.1001/jama.283.15.2008 10789670

[25] Pickkers P, Darmon M, Hoste E et al. Acute kidney injury in the critically ill: an updated review on pathophysiology and management. Intensive Care Med 2021;47:835–50. 10.1007/s00134-021-06454-7 34213593 PMC8249842

[26] Higgins JPT, Morgan RL, Rooney AA et al. A tool to assess risk of bias in non-randomized follow-up studies of exposure effects (ROBINS-E). Environ Int 2024;186:108602. 10.1016/j.envint.2024.108602 38555664 PMC11098530

[27] Fewtrell MS, Kennedy K, Singhal A et al. How much loss to follow-up is acceptable in long-term randomised trials and prospective studies? Arch Dis Child 2008;93:458. 10.1136/adc.2007.127316 18495909

[28] Wan X, Wang W, Liu J et al. Estimating the sample mean and standard deviation from the sample size, median, range and/or interquartile range. BMC Med Res Methodol 2014;14:135. 10.1186/1471-2288-14-135 25524443 PMC4383202

[29] Borenstein M, Hedges LV, Higgins JP et al. A basic introduction to fixed-effect and random-effects models for meta-analysis. Res Synth Method 2010;1:97–111. 10.1002/jrsm.12 26061376

[30] Deeks JJ, Higgins JPT, Altman DG; on behalf of the Cochrane Statistical Methods Group. Analysing data and undertaking meta-analyses. Cochrane Handbook for Systematic Reviews of Interventions 2019;241–84. 10.1002/9781119536604.ch10

[31] Sterne JA, Egger M. Funnel plots for detecting bias in meta-analysis: guidelines on choice of axis. J Clin Epidemiol 2001;54:1046–55. 10.1016/S0895-4356(01)00377-8 11576817

[32] McGuinness LA, Higgins JPT. Risk-of-bias VISualization (robvis): an R package and shiny web app for visualizing risk-of-bias assessments. Res Synth Methods 2021;12 55–61. 10.1002/jrsm.1411 32336025

[33] Carrero JJ, Fu EL, Sang Y et al. Discordances between creatinine- and cystatin C-based estimated GFR and adverse clinical outcomes in routine clinical practice. Am J Kidney Dis 2023;82:534–42. 10.1053/j.ajkd.2023.04.002 37354936

[34] He D, Gao B, Wang J et al. Differences between cystatin C- and creatinine-based estimated glomerular filtration rate and association with mortality and cardiovascular events: results from three cohorts of adults with diabetes. Nephrol Dial Transplant 2024;39:1322–32. 10.1093/ndt/gfae011 38317440

[35] He D, Gao B, Wang J et al. The difference between cystatin C- and creatinine-based estimated glomerular filtration rate and risk of diabetic microvascular complications among adults with diabetes: a population-based cohort study. Diabetes Care 2024;47:873–80. 10.2337/dc23-2364 38470988 PMC11043223

[36] Heo GY, Koh HB, Jung CY et al. Difference between estimated GFR based on cystatin C versus creatinine and incident atrial fibrillation: a cohort study of the UK Biobank. Am J Kidney Dis 2024;83:729–38.e1. 10.1053/j.ajkd.2023.11.004 38171411

[37] Herou E, Grubb A, Dardashti A et al. Reduced renal elimination of larger molecules is a strong predictor for mortality. Sci Rep 2022;12:17517. 10.1038/s41598-022-22433-4 36266435 PMC9584920

[38] Jonsson M, Åkesson A, Hommel A et al. Markers of renal function at admission and mortality in hip fracture patients—a single center prospective observational study. Scand J Clin Lab Invest 2021;81:201–7. 10.1080/00365513.2021.1884892 33606570

[39] Kim H, Park JT, Lee J et al. The difference between cystatin C- and creatinine-based eGFR is associated with adverse cardiovascular outcome in patients with chronic kidney disease. Atherosclerosis 2021;335:53–61. 10.1016/j.atherosclerosis.2021.08.036 34571286

[40] Ljungberg J, Johansson B, Bergdahl IA et al. Mild impairment of renal function (shrunken pore syndrome) is associated with increased risk for future surgery for aortic stenosis. Scand J Clin Lab Invest 2019;79:524–30. 10.1080/00365513.2019.1664761 31522562

[41] Malmgren L, McGuigan FE, Christensson A et al. Impaired selective renal filtration captured by eGFR(cysC)/eGFR(crea) ratio is associated with mortality in a population based cohort of older women. Sci Rep 2022;12:1273. 10.1038/s41598-022-05320-w 35075286 PMC8786879

[42] Potok OA, Ix JH, Shlipak MG et al. The difference between cystatin C- and creatinine-based estimated GFR and associations with frailty and adverse outcomes: a cohort analysis of the Systolic Blood Pressure Intervention Trial (SPRINT). Am J Kidney Dis 2020;76:765–74. 10.1053/j.ajkd.2020.05.017 32682697 PMC8896529

[43] Söderström E, Blind R, Wennberg P et al. Mild impairment of renal function (shrunken pore syndrome) is associated with increased risk of a future first-ever myocardial infarction in women. Scand J Clin Lab Invest 2021;81:438–45. 10.1080/00365513.2021.1941235 34237228

[44] Wu Z, Wang L, Li Y et al. Shrunken pore syndrome is associated with renal function decline in female patients with kidney diseases. Biomed Res Int 2022;2022:2177991. 10.1155/2022/2177991 35845935 PMC9283046

[45] Xhakollari L, Grubb A, Jujic A et al. The Shrunken pore syndrome is associated with poor prognosis and lower quality of life in heart failure patients: the HARVEST-Malmö study. ESC Heart Fail 2021;8:3577–86. 10.1002/ehf2.13485 34382359 PMC8497365

[46] Zhang LW, Luo MQ, Xie XW et al. Shrunken pore syndrome: a new and more powerful phenotype of renal dysfunction than chronic kidney disease for predicting contrast-associated acute kidney injury. J Am Heart Assoc 2023;12:e027980. 10.1161/JAHA.122.027980 36565177 PMC9973563

[47] Grubb A, Horio M, Hansson L-O et al. Generation of a new cystatin C–based estimating equation for glomerular filtration rate by use of 7 assays standardized to the international calibrator. Clin Chem 2014;60:974–86. 10.1373/clinchem.2013.220707 24829272

[48] Björk J, Grubb A, Larsson A et al. Accuracy of GFR estimating equations combining standardized cystatin C and creatinine assays: a cross-sectional study in Sweden. Clin Chem Lab Med 2015;53:403–14. 10.1515/cclm-2014-0578 25274955

[49] Inker LA, Eneanya ND, Coresh J et al. New creatinine- and cystatin C–based equations to estimate GFR without race. N Engl J Med 2021;385:1737–49. 10.1056/NEJMoa2102953 34554658 PMC8822996

[50] Pottel H, Hoste L, Dubourg L et al. An estimated glomerular filtration rate equation for the full age spectrum. Nephrol Dial Transplant 2016;31:798–806. 10.1093/ndt/gfv454 26932693 PMC4848755

[51] Almén MS, Björk J, Nyman U et al. Shrunken pore syndrome is associated with increased levels of atherosclerosis-promoting proteins. Kidney Int Rep 2019;4:67–79. 10.1016/j.ekir.2018.09.002 30596170 PMC6308389

[52] Cruz-Jentoft AJ, Bahat G, Bauer J et al. Sarcopenia: revised European consensus on definition and diagnosis. Age Ageing 2019;48:16–31. 10.1093/ageing/afy169 30312372 PMC6322506

[53] Sharma D, Hawkins M, Abramowitz MK. Association of sarcopenia with eGFR and misclassification of obesity in adults with CKD in the United States. Clin J Am Soc Nephrol 2014;9:2079–88. 10.2215/CJN.02140214 25392147 PMC4255396

[54] Jones SA, Fraser DJ, Fielding CA et al. Interleukin-6 in renal disease and therapy. Nephrol Dial Transplant 2015;30:564–74. 10.1093/ndt/gfu233 25011387

[55] van den Borne P, Quax PH, Hoefer IE et al. The multifaceted functions of CXCL10 in cardiovascular disease. Biomed Res Int 2014;2014:1. 10.1155/2014/893106 PMC4017714 24868552

[56] Gutiérrez OM, Mannstadt M, Isakova T et al. Fibroblast growth factor 23 and mortality among patients undergoing hemodialysis. N Engl J Med 2008;359:584–92. 10.1056/NEJMoa0706130 18687639 PMC2890264

[57] Åkesson A, Malmgren L, Leion F et al. Different ways of diagnosing selective glomerular hypofiltration syndromes such as shrunken pore syndrome and the associated increase in mortality. J Intern Med 2025;297:79–92. 10.1111/joim.20035 PMC11636450 39560353

