# Supplementary material for: Discordance between cystatin C–based and creatinine-based estimated glomerular filtration rate and health outcomes in adults: a systematic review and meta-analysis
Source: Clin Kidney J. 2025 Jan 8;18(3):sfaf003. doi: 10.1093/ckj/sfaf003 (PMC11997436; doi:10.1093/ckj/sfaf003)
Supplement: sfaf003_Supplemental_File [file sfaf003_supplemental_file.docx]

**CONTENTS**

Supplementary Table S1 Search terms for PubMed, Embase, and MEDLINE.

Supplementary Figure S1 Summarized risk of bias of included studies based on ROBINS-E scale.

Supplementary Figure S2 Detailed risk of bias of included studies based on ROBINS-E scale.

Supplementary Figure S3 Leave-one-out analyses of the association between eGFR discordance and mortality

Supplementary Figure S4 Funnel plot of the association between eGFR discordance and mortality

Supplementary Figure S5 Stratified meta-analyses on the association of eGFR discordance with mortality

Supplementary Figure S6 Leave-one-out analyses of the association between eGFR discordance and cardiovascular incidence, Fine-Gray competing risk model

Supplementary Figure S7 Funnel plot of the association between eGFR discordance and cardiovascular incidence, Fine-Gray competing risk model

Supplementary Figure S8 Meta-analyses of the association between eGFR discordance and cardiovascular incidence, Cox regression model

Supplementary Figure S9 Leave-one-out analyses of the association between eGFR discordance and cardiovascular incidence, Cox regression model

Supplementary Figure S10 Funnel plot of the association between eGFR discordance and cardiovascular incidence, Cox regression model

**Supplementary Table S1 Search terms for PubMed, Embase, and MEDLINE.**

| **Search number** | **Query** | **Results** |
| --- | --- | --- |
| 1 | Cystatin*[Title/Abstract] AND(creatin*[Title/Abstract] OR SCr[Title/Abstract] OR serum creatinine*[Title/Abstract] ) | 4,762 |
| 2 | difference*[Title/Abstract] OR discordance*[Title/Abstract] OR discrepanc*[Title/Abstract] OR divergenc*[Title/Abstract] OR disparit*[Title/Abstract] OR compar*[Title/Abstract] OR agreemen*[Title/Abstract] | 8,746,800 |
| 3 | Intra-individual*[Title/Abstract] OR intraindividual*[Title/Abstract] | 13,546 |
| 4 | #1 AND (#2 OR #3) | 2,778 |
| 5 | selective glomerular hypofilt*[Title/Abstract] OR SGHS[Title/Abstract] OR shrunken pore[Title/Abstract] | 80 |
| 6 | #4 OR #5 | 2,833 |
| 7 | "cohort studies"[MeSH] | 2,597,749 |
| 8 | "cross-sectional studies"[MeSH Terms] | 499,871 |
| 9 | #7 OR #8 | 3,004,862 |
| 10 | #6 AND #9 | 971 |
| 11 | "Animals"[Mesh] NOT "Humans"[Mesh] | 5,215,064 |
| 12 | #10 NOT #11 | 963 |
| 13 | editorial[pt] OR letter[pt] OR comment[pt] OR meta-analysis[pt] OR review[pt] | 5,640,329 |
| 14 | #12 NOT #13 | 950 |

**PubMed**

**Embase 1947-Present, updated daily**

| **Search number** | **Query** | **Results** |
| --- | --- | --- |
| 1 | (Cystatin* and (creatin* or SCr or serum creatinine*)).ti,ab. | 7984 |
| 2 | difference*.ti,ab. or discordance*.ti,ab. or discrepanc*.ti,ab. or divergenc*.ti,ab. or disparit*.ti,ab. or compar*.ti,ab. or agreement*.ti,ab. | 11893923 |
| 3 | Intra-individual*.ti,ab. or intraindividual*.ti,ab. | 17202 |
| 4 | 1 and (2 or 3) | 4920 |
| 5 | selective glomerular hypofilt*.ti,ab. or SGHS.ti,ab. or shrunken pore*.ti,ab. | 103 |
| 6 | 4 or 5 | 4989 |
| 7 | exp cohort studies/ | 1152510 |
| 8 | exp cross-sectional studies/ | 630718 |
| 9 | 7 or 8 | 1735479 |
| 10 | 6 and 9 | 983 |
| 11 | exp Animals/ not exp Humans/ | 6039093 |
| 12 | 10 not 11 | 981 |
| 13 | exp editorial/ or exp letter/ or exp meta-analysis/ or exp review/ | 5422522 |
| 14 | 12 not 13 | 971 |

**Ovid MEDLINE(R) ALL 1946 to April 28, 2024**

| **Search number** | **Query** | **Results** |
| --- | --- | --- |
| 1 | (Cystatin* and (creatin* or SCr or serum creatinine*)).ti,ab. | 4690 |
| 2 | difference*.ti,ab. or discordance*.ti,ab. or discrepanc*.ti,ab. or divergenc*.ti,ab. or disparit*.ti,ab. or compar*.ti,ab. or agreement*.ti,ab. | 8732743 |
| 3 | Intra-individual*.ti,ab. or intraindividual*.ti,ab. | 13371 |
| 4 | 1 and (2 or 3) | 2750 |
| 5 | selective glomerular hypofilt*.ti,ab. or SGHS.ti,ab. or shrunken pore*.ti,ab. | 81 |
| 6 | 4 or 5 | 2808 |
| 7 | exp cohort studies/ | 2598859 |
| 8 | exp cross-sectional studies/ | 500474 |
| 9 | 7 or 8 | 3006471 |
| 10 | 6 and 9 | 966 |
| 11 | exp Animals/ not exp Humans/ | 5215958 |
| 12 | 10 not 11 | 958 |
| 13 | exp editorial/ or exp letter/ or exp meta-analysis/ or exp review/ | 5350763 |
| 14 | 12 not 13 | 946 |

**Supplementary Figure S1 Summarized risk of bias of included studies based on ROBINS-E scale.**


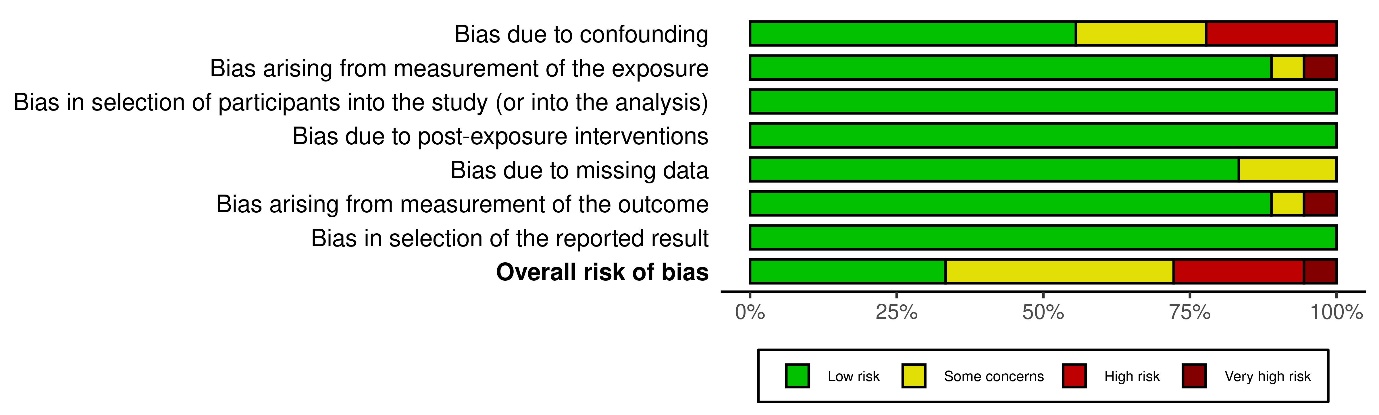


**Supplementary Figure S2 Detailed risk of bias of included studies based on ROBINS-E scale.**


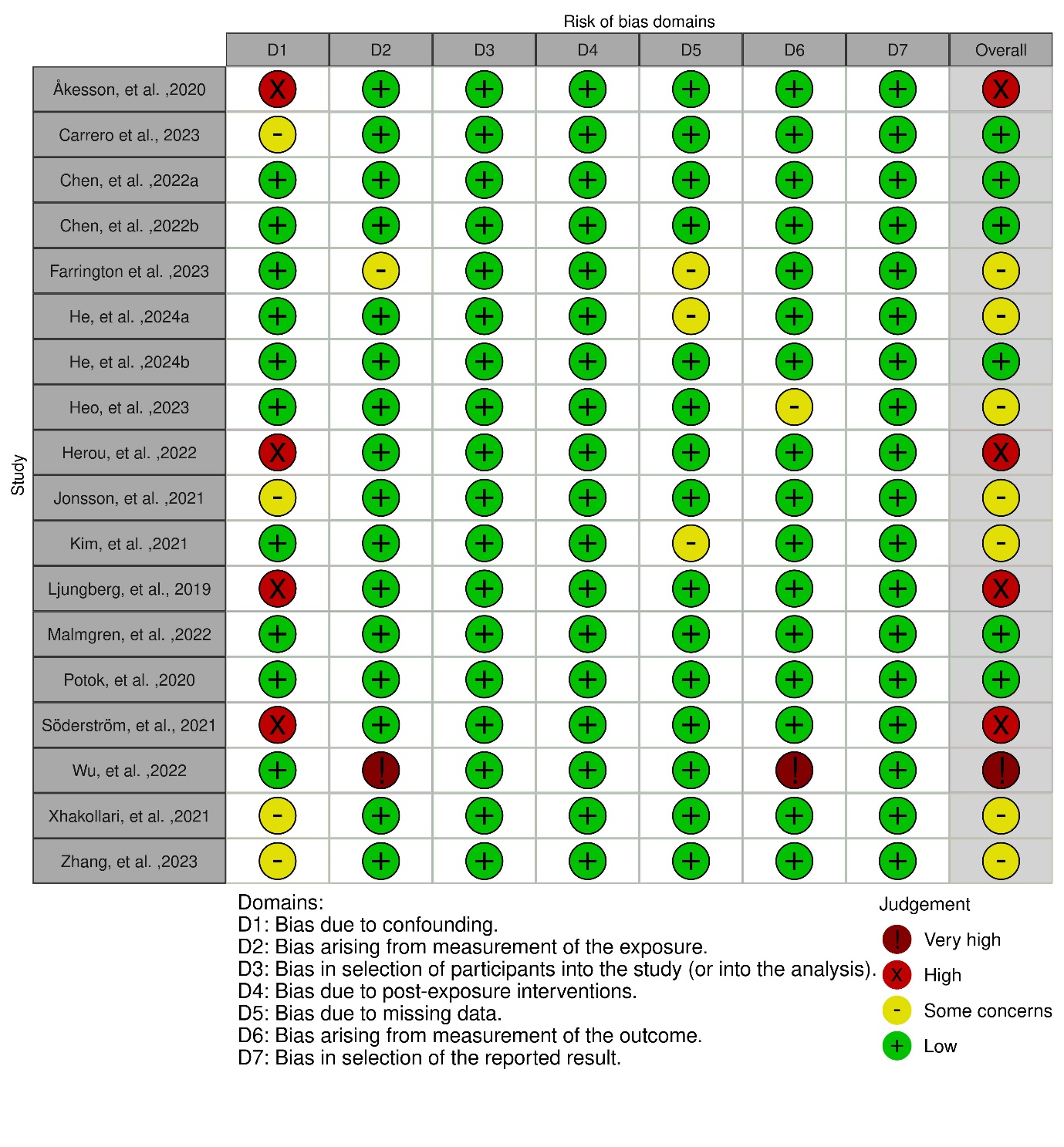


**Supplementary Figure S3 Leave-one-out analyses of the association between eGFR discordance and mortality**

**a. eGFRcys-eGFRcr < -15 ml/min/1.73m^2^, hazard ratio**

**b. eGFRcys-eGFRcr ≥ 15 ml/min/1.73m^2^, hazard ratio**

**c. eGFRcys-eGFRcr per +1 SD, hazard ratio**

**d. eGFRcys/eGFRcr≤0.6, hazard ratio**

**Supplementary Figure S4 Funnel plot of the association between eGFR discordance and mortality**

**a. eGFRcys-eGFRcr < -15 ml/min/1.73m^2^**

**b. eGFRcys-eGFRcr ≥ 15 ml/min/1.73m^2^**

**c. eGFRcys-eGFRcr per +1 SD**

**d. eGFRcys/eGFRcr≤0.6**

**Supplementary Figure S5 Stratified meta-analyses on the association of eGFR discordance with mortality**

**a. eGFRcys-eGFRcr < -15 ml/min/1.73m^2^, stratified by C-reactive protein level**

**b. eGFRcys/eGFRcr≤0.6, stratified by C-reactive protein level**

**c. eGFRcys/eGFRcr≤0.6, stratified by obesity status**

**Supplementary Figure S6 Leave-one-out analyses of the association between eGFR discordance and cardiovascular incidence, Fine-Gray competing risk model**

**a. eGFRcys-eGFRcr < -15 ml/min/1.73m^2^, subharzard ratio**

**b. eGFRcys-eGFRcr ≥ 15 ml/min/1.73m^2^, subharzard ratio**

**c. eGFRcys-eGFRcr per +1 SD, subharzard ratio**

**d. eGFRcys/eGFRcr<0.6, subharzard ratio**

**Supplementary Figure S7 Funnel plot of the association between eGFR discordance and cardiovascular incidence, Fine-Gray competing risk model**

**a. eGFRcys-eGFRcr < -15 ml/min/1.73m^2^**

**b. eGFRcys-eGFRcr ≥ 15 ml/min/1.73m^2^**

**c. eGFRcys-eGFRcr per +1 SD**

**d. eGFRcys/eGFRcr<0.6**

**Supplementary Figure S8 Meta-analyses of the association between eGFR discordance and cardiovascular incidence, Cox regression model**

**a. eGFRcys-eGFRcr < -15 ml/min/1.73m^2^**

**b. eGFRcys-eGFRcr ≥ 15 ml/min/1.73m^2^**

**c. eGFRcys-eGFRcr per +1 SD**

**d. eGFRcys/eGFRcr<0.6**

**Supplementary Figure S9 Leave-one-out analyses of the association between eGFR discordance and cardiovascular incidence, Cox regression model**

**a. eGFRcys-eGFRcr < -15 ml/min/1.73m^2^**

**b. eGFRcys-eGFRcr ≥ 15 ml/min/1.73m^2^**

**c. eGFRcys-eGFRcr per +1 SD**

**d. eGFRcys/eGFRcr<0.6**

**Supplementary Figure S10 Funnel plot of the association between eGFR discordance and cardiovascular incidence, Cox regression model**

**a. eGFRcys-eGFRcr < -15 ml/min/1.73m^2^**

**b. eGFRcys-eGFRcr ≥ 15 ml/min/1.73m^2^**

**c. eGFRcys-eGFRcr per +1 SD**

**d. eGFRcys/eGFRcr<0.6**
